# A Novel Spectral Annotation Strategy Streamlines Reporting of Mono-ADP-ribosylated Peptides Derived from Mouse Liver and Spleen in Response to IFN-γ

**DOI:** 10.1016/j.mcpro.2021.100153

**Published:** 2021-09-28

**Authors:** Shiori Kuraoka, Hideyuki Higashi, Yoshihiro Yanagihara, Abhijeet R. Sonawane, Shin Mukai, Andrew K. Mlynarchik, Mary C. Whelan, Michael O. Hottiger, Waqas Nasir, Bernard Delanghe, Masanori Aikawa, Sasha A. Singh

**Affiliations:** 1 Center for Interdisciplinary Cardiovascular Sciences, Division of Cardiovascular Medicine, Department of Medicine, Brigham Women's Hospital, Harvard Medical School, Boston, Massachusetts, USA; 2 Center for Excellence in Vascular Biology, Division of Cardiovascular Medicine, Brigham Women's Hospital, Harvard Medical School, Boston, Massachusetts, USA; 3 Department of Molecular Mechanisms of Disease, University of Zurich, Zurich, Switzerland; 4 Thermo Fisher Scientific (Bremen) GmbH, Bremen, Germany; 5 Channing Division of Network Medicine, Department of Medicine, Brigham Women's Hospital, Harvard Medical School, Boston, Massachusetts, USA

**Keywords:** PARP14/ARTD8, inflammation, mass spectrometry, proteomics, posttranslational modification, ADPr, ADP-ribosyl, ARTD, ADP-ribosyl transferase, CID, collision-induced dissociation, ECD, electron capture dissociation, ETD, electron transfer dissociation, FDR, false discovery rate, HCD, higher-energy-induced dissociation, MARylated, mono-ADP-ribosylated, PARylated, poly-ADP-ribosylated, PSM, peptide-spectrum match, PTM, posttranslational modification

## Abstract

Mass-spectrometry-enabled ADP-ribosylation workflows are developing rapidly, providing researchers a variety of ADP-ribosylome enrichment strategies and mass spectrometric acquisition options. Despite the growth spurt in upstream technologies, systematic ADP-ribosyl (ADPr) peptide mass spectral annotation methods are lacking. HCD-dependent ADP-ribosylome studies are common, but the resulting MS2 spectra are complex, owing to a mixture of *b/y*-ions and the *m/p*-ion peaks representing one or more dissociation events of the ADPr moiety (*m*-ion) and peptide (*p*-ion). In particular, *p*-ions that dissociate further into one or more fragment ions can dominate HCD spectra but are not recognized by standard spectral annotation workflows. As a result, annotation strategies that are solely reliant upon the *b/y*-ions result in lower spectral scores that in turn reduce the number of reportable ADPr peptides. To improve the confidence of spectral assignments, we implemented an ADPr peptide annotation and scoring strategy. All MS2 spectra are scored for the ADPr *m*-ions, but once spectra are assigned as an ADPr peptide, they are further annotated and scored for the *p*-ions. We implemented this novel workflow to ADPr peptides enriched from the liver and spleen isolated from mice post 4 h exposure to systemic IFN-γ. HCD collision energy experiments were first performed on the Orbitrap Fusion Lumos and the Q Exactive, with notable ADPr peptide dissociation properties verified with CID (Lumos). The *m*-ion and *p*-ion series score distributions revealed that ADPr peptide dissociation properties vary markedly between instruments and within instrument collision energy settings, with consequences on ADPr peptide reporting and amino acid localization. Consequentially, we increased the number of reportable ADPr peptides by 25% (liver) and 17% (spleen) by validation and the inclusion of lower confidence ADPr peptide spectra. This systematic annotation strategy will streamline future reporting of ADPr peptides that have been sequenced using any HCD/CID-based method.

## Highlights

• An annotation method to identify and score ADP-ribosyl (ADPr) peptide MS2 spectra.
• The m-ion score monitors the dissociation of the ADPr modification.
• The p-ion score monitors the dissociation of the peptide plus residual ADPr fragment.
• The p-ion score increased reportable ADPr peptide numbers in mouse tissues.

## Graphical abstract

**Figure:**
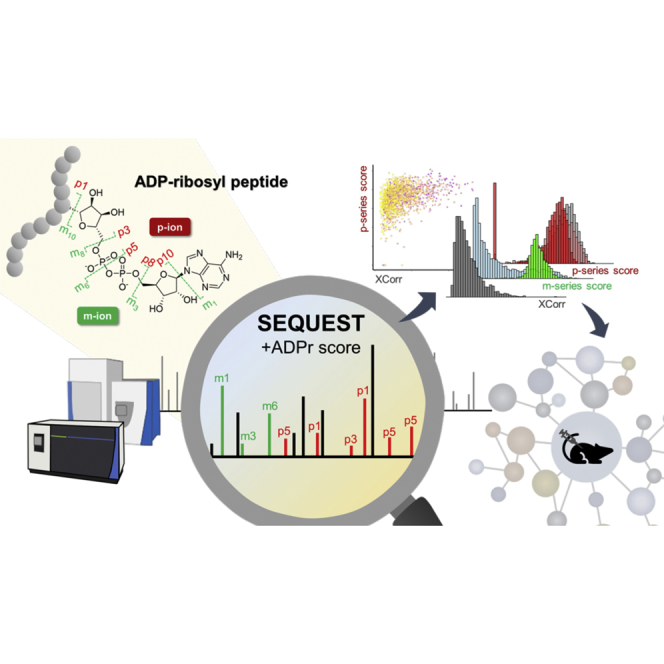

## In Brief

ADP-ribosyl peptides exhibit complex HCD-derived MS2 spectra that comprise peaks corresponding to the complete or partial dissociation of the ADP-ribosyl moiety itself (m-ions) and peaks corresponding to the residual modification on the intact peptide (P-ion) that in turn undergoes further dissociation to produce peptide fragments (p-ions). Our novel workflow annotates and scores these unique ADP-ribose fragments not recognized by standard annotation methods. This workflow increased the number of reportable ADP-ribosyl peptides identified in a mouse model for acute proinflammatory response.

ADP-ribosylation is a posttranslational modification (PTM) that is catalyzed by the polyadenosine diphosphate-ribose polymerase (PARP) enzyme family, also referred to as the diphtheria toxin-like ADP-ribosyl transferases (ARTDs) (1). The PARPs catalyze the transfer of the ADP-ribosyl (ADPr) moiety of NAD to proteins targets, whose amino acid acceptor sites have been identified as primarily aspartate, glutamate, lysine, arginine, and serine but also threonine, tyrosine, histidine, and cysteine (2, 3, 4, 5). The ubiquity of protein ADP-ribosylation is becoming more apparent as the mass-spectrometry-based workflows used to identify ADP-ribosylated proteins continue to improve. ADP-ribosylome studies are of growing interest since the PARP enzymes are implicated in a variety of cellular functions, such as oxidative stress and DNA repair (6), RNA biology (7), and host–pathogen interactions and inflammation (7, 8). As a consequence of their broad roles in biology, the PARP substrate pool is likely vaster than what has already been reported (8).

There is an assortment of wet lab and mass spectrometry workflows available to study ADP-ribosylation. Proteins can be mono-ADP-ribosylated (MARylated) or poly-ADP-ribosylated (PARylated) (9), but only hydrolyzed forms of the PTM, for example, the conversion of PAR to MAR peptides using poly-ADP-ribose glycohydrolase (PARG) (2, 10, 11) and the conversion of PAR/MAR to a phosphoribose using a phosphodiesterase (12), are conducive to mass spectrometry. As of late, MARylation-based enrichment studies are gaining traction since ADPr peptides produce diagnostic MS2 fragment ions that can be used to increase confidence that spectra contain modified peptides (13, 14, 15). The ADP-ribose is labile with collision-induced dissociation (CID) and higher-energy-induced dissociation (HCD), producing lower mass fragments from the ADP-ribose moiety (*m*-ions) and the complementary peptide plus remaining ADP-ribose fragment (*p*-ions) (13).

Despite the convenience of diagnostic ions, ADPr peptides exhibit complex fragmentation properties, requiring more than one dissociation method for their annotation. CID, for instance, produces too few backbone *b/y*-ions for peptide identification (13) and is therefore not employed for routine ADPr peptide sequencing. On the other hand, sequential dissociation provided by HCD increases the prevalence of backbone fragments (14), but also promotes dissociation of the peptide precursor *p*-ions into fragment *p*-ions. This sequential dissociation increases the complexity of MS2 spectra to be interpreted since MS2 sequencing algorithms do not readily recognize *p*-ions, other than the *p*-ion corresponding to the complete loss of the ADPr modification. Candidate ADPr spectra and acceptor sites are validated by either manual inspection or by supplemented scripts that confirm the presence of *p*-ions (3, 11). Electron capture and transfer dissociation (ECD, ETD) (13, 16) retain the intact ADP-ribose on the acceptor amino acid, which is amenable to acceptor site localization. However, these dissociation methods are slower than HCD, reducing the number of candidate ADPr peptide spectra to be analyzed. Moreover, they work best with highly charged ADPr peptides (14). Very recently, infrared photo-activation ionization (AI)-ETD was demonstrated to outperform HCD as a supplemented activation method to analyze MARylated peptides (17). Studies enriching MARylated peptides thus leverage the pros of each method and employ various dissociations strategies or combinations thereof (*e.g.*, EThcD) to maximize ADPr peptide sequencing (2, 11, 14).

We previously reported the ADP-ribosylome of the human macrophage cell-like line THP-1 stimulated with or without the proinflammatory cytokine IFN-γ (11). Our particular interests in macrophage ADP-ribosylation stemmed from our earlier report describing PARP14 and PARP9 as novel IFN-γ inducible proteins and potential regulators of proinflammatory activation (18). IFN-γ-stimulated THP-1 cells also resulted in an increase in the ADP-ribosylation status of PARP14 and PARP9 and several ribosomal and heat shock proteins (11). In the THP-1 ADP-ribosylome study, we employed the MARylation enrichment strategy. To further enrich for ADPr peptides within the mass spectrometer, we applied an *m*-ion-dependent triggered strategy that employed a fast (lower-end resolution, higher-end collision energy) HCD scan to screen for diagnostic *m*-ions that if detected was followed by alternating HCD (higher-end resolution, lower-end collision energy) and EThcD analysis scans (11, 14). This specific acquisition strategy therefore ensured that each precursor ADPr peptide would benefit from both HCD and EThcD dissociation methods.

Despite the prevalence of ADPr diagnostic *m*-ions and *p*-ions in the MS2 spectra, we and others have been limited to standard peptide scoring algorithms and confidence scores to identify and report ADPr peptides. However, there is great potential in implementing a fragment *p*-ion scoring strategy to supplement *b/y*-ion annotation methods. Fragment *p*-ions are prevalent in HCD MS2 spectra but represent complex peaks owing to dissociation of both the backbone peptide and the ADPr PTM (15). Nonetheless, fragment *p*-ions harbor information for peptide and amino acid acceptor site identification but are not considered in standard peptide scoring workflows, thereby underutilizing the full potential of ADPr peptide spectral features.

In this current study, we have developed a novel ADPr spectrum annotation workflow that overcomes the caveats of relying on standard spectral annotation algorithms to score ADPr spectra. The ADPr annotation workflow comprises an *m*-ion series score and a *p*-ion series score that are used to identify and validate candidate ADPr MS2 spectra, respectively. We applied this new ADPr annotation workflow to analyze the ADP-ribosylome from the liver and spleen isolated from mice post an IFN-γ-induced pro-inflammatory response. Using a subset of liver samples, we first performed several mass spectrometric acquisition optimization trials, performed on the Orbitrap Fusion Lumos (HCD, CID) and Q Exactive (HCD), which monitored the impact of the *m*-ion and *p*-ion series scores on the outcome of ADPr spectral annotation. Once optimal spectral annotation strategies and confidence thresholds were established, we completed the ADP-ribosylome analysis of the IFN-γ-challenged mouse tissues.

## Experimental Procedures

### Experimental Design and Statistical Rationale

**Fig. 1:**
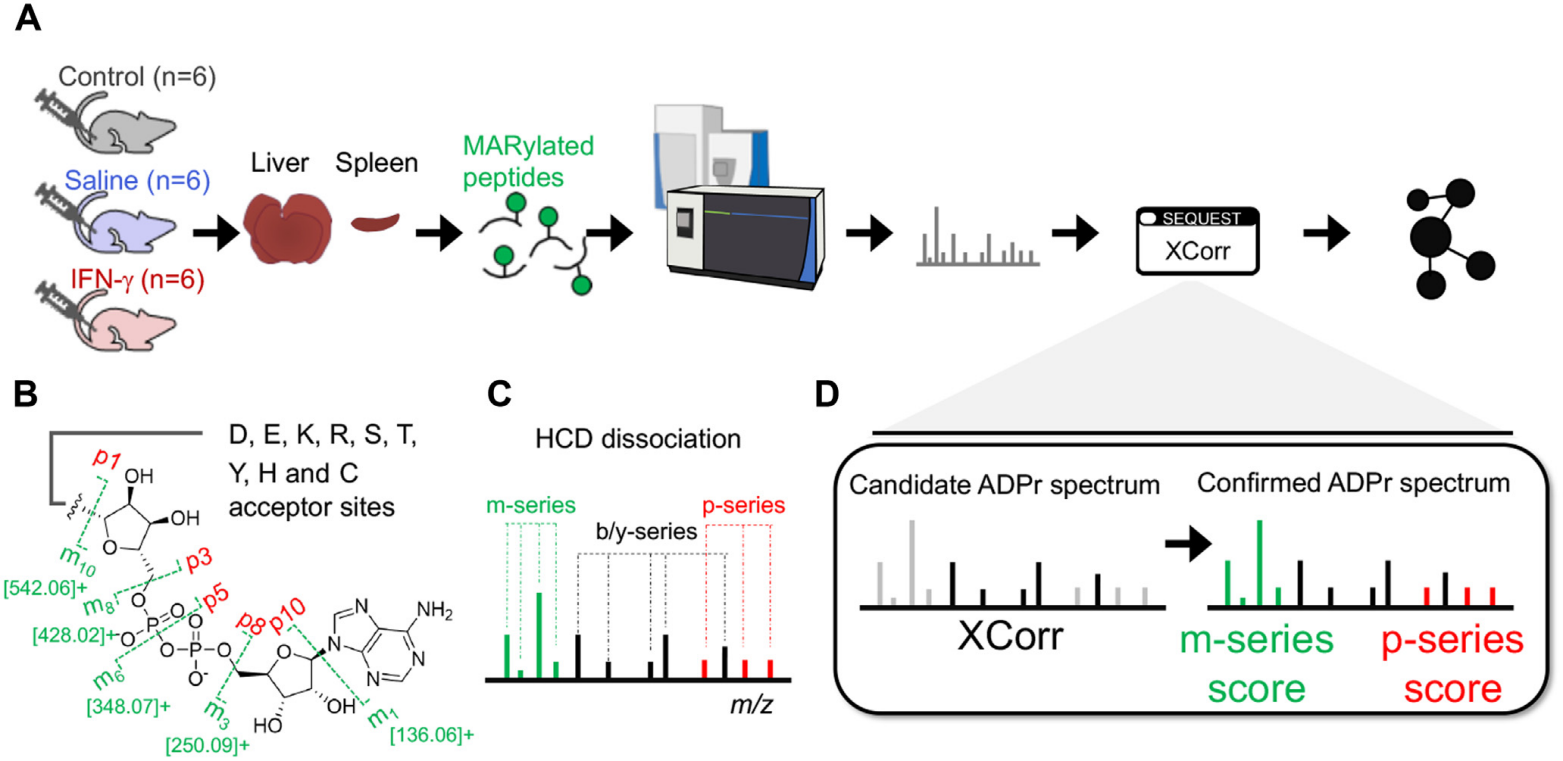
**ADP-ribosylome bench to spectral annotation workflow**. *A*, IFN-γ induced ADP-ribosylome study. Ten-week old sibling mice were injected with saline or IFN-γ, or nothing at all (no treatment control) (n = 6 per treatment group). Spleen and liver organs were harvested for subsequent mono-ADPr (MAR)ylated peptide enrichment. The samples were analyzed by the Q Exactive and/or the Lumos, and the spectra were annotated using SEQUEST *via* Proteome Discoverer 2.4. *B*, the ADPr structure and fragment ions (modification ion, *m*-ion (and [MH]+ values); the complementary peptide ion, *p*-ion). Amino acid acceptor sites are indicated. *C*, schematic of a typical HCD-generated ADPr peptide spectrum. *D*, candidate ADPr spectra identified and scored (XCorr) by the search engine (SEQUEST-HT) are then supplemented with m-ion and p-ion series scores to help validate the assignments.

A pilot IFN-γ (*versus* saline) dosing and time course study was done to establish optimal treatment conditions (n = 2 mice per condition and dose and time point for the liver and spleen). The remaining liver samples were used to prepare a pilot ADPr peptide pool to perform collision energy experiments; and spleen pilot samples were used to perform targeted MS2 (tMS2) validations. For the main mouse infusion experiments performed in this study (Fig. 1*A*), a total of seven male mice per group (no treatment control, saline, and IFN-γ) were compared for RT-PCR, but n = 6 were used per group for proteomics. Male mice alone were chosen to eliminate potential variation due to sex. Protein yields (10 mg) from liver portions were sufficient to analyze each liver separately (n = 6 per group); however, two spleens had to be pooled resulting (n = 3 per group) to be compared. Pooling spleens was supported by the verification that IFN-γ elicited a response in all six mice, as determined by monitoring prototypical IFN-γ-responsive genes (presented in the latter half of the study).

### Mouse Infusions

All animal procedures used in this study were approved by and performed in compliance with Beth Israel Deaconess Medical Center's Institutional Animal Care and Use Committee (protocol #: 021-2017). Male C57BL/6J mice (10 weeks old, The Jackson Laboratory, Bar Harbor, ME, Cat# 000664) were treated with either IFN-γ (100 K units/mouse, 1.0 ml, R&D Systems, Minneapolis, MN, Cat# 485-MI-100) or saline (1.0 ml, Owens & Minor, Mechanicsville, VA, Cat# 85583) through intraperitoneal injection, and then an analgesic (buprenorphine, 1.2 mg/kg, Henry Schein Animal Health, Melville, NY, Cat# 1217793) was given by subcutaneous injection. At 4 h they were sacrificed in a CO_2_ chamber. The mice were perfused with 10 ml of a PARP/PARG inhibitory buffer [1.0 μM PJ-34 (Millipore Sigma, Cat# 528150), 250 nM ADP-HPD (Millipore Sigma, Cat# 118415), phosphate buffered saline (PBS, Lonza, Basel, Switzerland, Cat# 17-517Q)] through a left ventricle. Liver and spleen were collected and gently washed with PBS. Each tissue was divided into two, with the majority for *Western blot analysis* and *Proteolysis for proteomic analysis* (see below) and a smaller portion sufficient for Real-Time PCR (below).

### Tissue Harvest and Homogenization

Each tissue was transferred to a Precellys 2 ml tube from the Soft Tissue Homogenizing Ceramic Beads Kit (Bertin Technologies, Montigny-le-Bretonneux, France, Cat# CK14). TRIzol (0.5 ml, Thermo Fisher Scientific, Waltham, MA, Cat# 15596-018) was added to tissues for real-time-PCR. A modified RIPA buffer [0.5 ml, 50 mM Tris-HCl pH 7.4 (Boston BioProducts, Ashland, MA, Cat# BM-327), 0.4 M NaCl (Sigma-Aldrich, St Louis, MO, Cat# S9888), 1.0 mM EDTA (Boston Bio Products, Cat# BM-150), 1.0% nonidet P-40 (Sigma-Aldrich, Cat# 74385), 0.1% sodium deoxycholate (Sigma-Aldrich, Cat# D6750), 40 μM PJ34, 1.0 μM ADP-HPD, protease inhibitor cocktail (Sigma-Aldrich, Cat# P8340), phosphatase inhibitor (Sigma-Aldrich, Cat# 4906845001)] (10) were added to tissues for protein analysis. Tissues were homogenized in a Precellys 24 Tissue Homogenizer (Bertin Technologies, Cat# P000669-PR240-A) using three 10 s cycles at 5000 rpm that were then cooled on ice for 15 min. The tissue debris was then removed by centrifugation (3000 rpm, 4 °C, 5 min).

### Experiments to Confirm IFN-γ Elicited a Proinflammatory Response in Mice

#### Real-Time PCR

Reverse transcription was performed using qScript cDNA Synthesis Kit (QuantaBio). The mRNA expression was determined by 7900 HT Fast Real time PCR System (Thermo Fisher Scientific). Master mix for real-time PCR was PerfeCTa qPCR FastMix II, ROX (VWR International, Radnor, PA, Cat# 97065-998), and TaqMan probes were used as follows: human GAPDH (Hs02758991_g1, Life Technologies), human PARP14 (Hs00981511_m1), human IL-1β (Hs00174097_m1), human CCL2 (Hs00234140_m1), human CXCL9 (Hs00171065_m1), human CXCL10 (Hs01124252_g1), human CXCL11 (Hs04187682_g1). The expression levels were normalized to human GAPDH (Hs02758991_g1).

#### Western Blot Analysis

The protein amount was determined using a Pierce BCA Protein Assay Kit (Thermo Fisher Scientific, Cat# 23225). One milligram of protein (diluted into 100 ul modified RIPA buffer) was mixed with 20 μl 6× SDS-Sample Buffer (Boston Bio Products, Cat# BP-111R) and boiled at 95 ^°^C for 5 min. The denatured samples were separated by SDS-PAGE [8.0% acrylamide (Boston Bio Products, Cat# BAC-30PA), BAC-30PA (Boston Bio Products, Cat# BP-90), stacking buffer (Boston Bio Products, Cat# BP-95), N,N,N′,N′-tetramethylethylenediamine (TEMED, Sigma-Aldrich, Cat# 1610801), ammonium persulfate (Sigma-Aldrich, Cat# A3678-25G)] and transferred to nitrocellulose membranes (Bio-Rad Laboratories, Hercules, CA, Cat# 1620112). The membranes were blocked with 2.5% nonfat dry milk (Santa Cruz Biotechnology, TX, Cat# sc-2325) in 1× tris-buffered saline with 0.1% Tween 20 (TBST, Boston BioProducts, MA, Cat# IBB-181). The following primary antibodies were used: a human ARTD8/PARP14 antibody (1:1000, Santa Cruz Biotechnology, Cat# sc-377150); a pan ADP-ribose reagent (1:300, Millipore Sigma, Cat# MABE1016); a β-actin antibody (1:5000, Novus Biologicals, LLC, CO, Cat# NB600-501). The secondary antibodies were anti-mouse peroxidase conjugate (1:5000, Sigma-Aldrich, Cat# A4416-1ML) and anti-rabbit peroxidase conjugate (1:5000, Sigma-Aldrich, Cat# A0545-1ML) as required for the primary antibodies and detected using Clarity Western ECL blotting substrate (Bio-Rad Laboratories, Cat# 1705060) and imaged using ImageQuant LAS 4000 (GE Healthcare).

### Proteolysis Steps

For protein precipitation, acetone (Fisher Scientific, Hampton, NH, Cat# A949-1) was added to the tissue homogenates (10) and then resuspended in a denaturation buffer (6.0 M urea [Sigma-Aldrich, Cat# U4884], 2.0 M thiourea [Sigma-Aldrich, Cat# T7875], 10 mM HEPES [Boston BioProducts, Cat# BBH-75-K]). The protein amount was determined by a Pierce 660 nm Protein Assay Reagent (Thermo Fischer Scientific, Cat# 22660). Proteins (5.0–10 mg, two spleen tissues were merged) were reduced in 1.0 mM dithiothreitol (DTT, Thermo Fisher Scientific, Cat# 20290) and alkylated in 5.5 mM chloroacetamide (Sigma-Aldrich, Cat# C0267). Proteolysis was performed with LysC for 4 h, followed by trypsin (following the recommended protocols of the Trypsin/Lys-C Mix, VWR International, Cat# V5072) in 20 mM ammonium bicarbonate (Sigma-Aldrich, Cat# 09830) overnight. The peptides were desalted using Sep-Pak C18 Classic Cartridge (Waters, Milford, MA, Cat# WAT051910) by following the manufacturer's instructions. Using a Concentrator plus complete system (Eppendorf AG, Hamburg, Germany, Cat# 5305000304), the peptide sample was reduced to a final volume of 0.8 ml of affinity precipitation buffer (50 mM Tris-HCl pH 7.4, 10 mM MgCl_2_ [Sigma-Aldrich, Cat# 63069], 250 μM DTT, 50 mM NaCl). Peptide amount was determined by using a NanoDrop2000 Spectrophotometer at 280 nm (Thermo Fisher Scientific). One hundred microgram input peptide was set aside to measure the proteome, whereas 3.0–4.0 mg of peptide was used for the eAf1521 enrichment protocol.

### eAf1521 Macrodomain Removal and Peptide Recovery

We used the recently engineered *Archaeoglobus fulgidus* macrodomain (eAf1521) (19) to enrich MARylated peptides. Expression and purification steps were done according to a published protocol (10). However, this study includes further optimization of this protocol, by removing the eAf1521 from the enriched ADPr peptides eAf1521 (47 kDa) done using a molecular weight cutoff (MWCO) filter cartridge, the Microcon-30 kDa Centrifugal Filter Unit (Millipore Sigma, Cat# MRCF0R03).

MWCO cartridges 1–4 (supplemental Fig. S1) were equilibrated by passing 300 μl of 20% acetonitrile (Fisher Scientific, Cat# A955-1) /LC-MS-grade water (Fisher Scientific, Cat# W6-1) twice, 300 μl of 0.1 mol/l NaOH (Honeywell International, Charlotte, NC, Cat# 71463 Fluka) twice, 300 μl of LC/MS-grade water twice, and 300 μl of 0.15% trifluoroacetic acid (TFA, Sigma Aldrich, Cat# 302031) three times (14,000 rpm, 5 min). MWCO cartridge 5 was equilibrated by following a published protocol (2).

As a proof-of-concept that the eAf1521 is retained by the cartridge while peptides are recovered, we performed the following experiments. The free eAf1521 macrodomain (from 50 μl of the conjugates, (10)) or Pierce HeLa Protein Digest Standard (1.0 μg, Thermo Fisher Scientific, Cat# 88328) was used as input into each equilibrated MWCO cartridge. The input samples were diluted in 0.15% TFA (total volume 300 μl) and passed through each cartridge (14,000 rpm, 10 min). An additional 0.15% TFA (300 μl) was passed through (14,000 rpm, 10 min), and then the flow-through and retained fractions were collected to new tubes (low protein binding collection tubes, Thermo Fisher Scientific, Cat# 90411). The samples were dried down using a tabletop speed vacuum (60 °C, 2 h, Thermo Fisher Scientific, Cat# SPD1010). The flow-through and retained fractions for the eAf1521 experiment were resuspended in 1× SDS-sample buffer (Boston Bio Products, Cat# BP-111R) and boiled at 95 °C for 5 min for SDS-PAGE (Mini-PROTEAN TGX Precast Protein Gels, Bio-Rad Laboratories, Hercules, CA, Cat# 4561094, Precision Plus Protein Dual Color Standards, Bio-Rad Laboratories, Cat# 161-0394, Tris-Glycine-SDS Running Buffer, Boston BioProducts, Cat# BP-150). The gel was stained with Bio-Safe Coomassie Stain (Bio-Rad Laboratories, Cat# 1610786) by following the instructions. The flow-through and retained fractions for HeLa standard peptides were resuspended in loading buffer (5.0% acetonitrile [Fisher Scientific, Cat# A955-1], 0.5% formic acid [Thermo Fisher Scientific, Cat# 28905] in water [Fisher Scientific, Cat# W6-1]) for LC-MS/MS analysis. The Micron cartridge peptide flow-through is most similar to input, indicating optimal and ideal recovery of peptides (supplemental Fig. S2).

The MS2 data from the HeLa digests were queried against the human UniProt database (downloaded on November 2018; 155,133 entries) using the SEQUEST-HT search algorithm, *via* the Proteome Discoverer (PD) Package (version 2.2, Thermo Fisher Scientific), using a 10 ppm tolerance window in the MS1 search space and a 0.02 Da fragment tolerance window for HCD. Trypsin (full) was set as the digestion enzyme, allowing up to four missed cleavages and a minimum peptide length of six amino acids. Oxidation of methionine was set as a variable modification and carbamidomethylation of cysteine was set as a fixed modification. The peptide false discovery rate (FDR) was calculated using Percolator provided by PD, and peptides were filtered based on a 1.0% FDR. Peptides assigned to a given protein group, and not present in any other protein group, were considered as unique. Consequently, each protein group is represented by a single master protein (PD grouping feature). Master proteins with two or more unique peptides were used for precursor ion intensity-based quantification. Statistical analysis was performed on Qlucore (https://www.qlucore.com/).

### eAf1521-Dependent Enrichment of MARylated Peptides

Expression and purification of the eAf1521 macrodomain were done according to a published protocol (10). The peptide mixture was treated with PARG overnight (1.0 μg PARG per 1.0 mg peptide, Creative BioMart, Shirley, NY, Cat# PARG-31H) to obtain only MARylated peptides (2), and the peptides were enriched using the macrodomain affinity pull-down as described previously (10). Eighty percent of eluted ADPr peptides were processed using the MWCO filtration step using the Microcon-30 kDa Centrifugal Filter Unit, Millipore Sigma, Cat# MRCF0R03 (see eAf1521 Macrodomain Removal and Peptide Recovery). The peptides were desalted using Oasis HLB cartridge (10 mg [Waters, 1 cc, Cat# 186008055] for the input peptides; 30 mg [Waters, 1 cc, Cat# WAT094225] for the MARylated peptides) by following its instruction and suspended in loading buffer (5.0% acetonitrile [Fisher Scientific, Cat# A955-1], 0.5% formic acid [Thermo Fisher Scientific, Cat# 28905] in water [Fisher Scientific, Cat# W6-1]) for LC-MS/MS analysis.

#### LC-MS/MS

ADPr (MARylated) peptides from control, saline, and IFN-γ elicited mouse tissues (spleen and liver) were analyzed using the Orbitrap Fusion Lumos fronted with an EASY-Spray Source, coupled to an Easy-nLC1000 HPLC pump (Thermo Fisher Scientific), and the Q Exactive Orbitrap (+Easy-nLC1000) fronted with a Nanospray FLEX ion source (Thermo Fisher Scientific).

##### Lumos Collision Energy Experiments

A pool of mouse liver ADPr peptides from the pilot study were subjected to a dual column setup: an Acclaim PepMap RSLC C18 trap column, 75 μm 20 mm (Thermo Fisher Scientific, Cat# 164261); and an EASY-Spray LC Column, 75 μm × 250 mm (Thermo Fisher Scientific, Cat# ES802A). The analytical gradient for the ADPr peptide pool was run at 300 nl/min from 5 to 21 % Solvent B (acetonitrile/0.1% formic acid) for 50 min, followed by 10 min of 21 to 30% Solvent B, and another 10 min of a jigsaw wash (alternating between 5 and 95% Solvent B) to clean the column. Solvent A was water/0.1% formic acid. The instrument was set to 120 K resolution, and the top N precursor ions (within a scan range of *m/z* 400–1500) in 3 s cycle time were subjected to MS/MS. Dynamic exclusion was enabled (60 s), the isolation width was *m/z* 1.2, and the resolution was 120 K (automatic gain control, AGC, 1.0e4). HCD collision energies were set to 20%, 24%, 26%, 28%, 30%, 32%, or 34%. The CID collision energy settings were 20%, 24%, 26%, 28%, 30%, 32%, 34%, 36%, and 40%.

##### Q Exactive Collision Energy Experiments

A pool of mouse liver ADPr peptides from the pilot study were subjected to a dual column setup: an Acclaim PepMap RSLC C18 trap column, 75 μm × 20 mm (Thermo Fisher Scientific, Cat# 164261); and an Acclaim PepMap 100 C18 HPLC column, 75 μm × 250 mm (Thermo Fisher Scientific, Cat# 164941). The analytical gradient for the ADPr peptide pool was run at 300 nl/min from 5 to 21 % Solvent B (acetonitrile/0.1% formic acid) for 50 min, followed by 10 min of 21–30% Solvent B, and another 10 min of a jigsaw wash. The instrument was set to 70 K resolution (AGC target, 3e6), and the top ten precursor ions (within a scan range of *m/z* 400–1500) were subjected to HCD isolation width *m/z* 1.6, dynamic exclusion enabled (60 s), and resolution set to 140 K (AGC target, 5e4). The HCD collision energies were set to 20%, 24%, 26%, 28%, 30%, 32%, or 34%.

##### Lumos-Dependent Analysis of IFN-γ-Elicited Mouse Liver and Spleen ADPr Peptides

The ADP-ribose product ion triggered method was applied (11, 20). The chromatographic conditions were the same as the Lumos collision energy experiments. The *m*-ion product scan employed data-dependent HCD acquisition (collision energy 30% ± 2.5%, isolation width *m/z* 1.2, scan range *m/z* 120–445 to capture only the *m*-ions, and resolution set to 30 K). When two or more ADP-ribose fragment ions (*m/z* 136.0623, 250.0940, 348.0709, and 428.0372) were detected, alternating HCD (CE 27.5% +/- 2.5%; resolution 120 K) and EThcD (calibrated charge-dependent ETD parameters enabled, supplemental activation collision energy 22.5%, and resolution 120 K) scans were triggered. Each ADPr peptide sample was injected five times (n = 6 for control, saline and IFN-γ liver; n= 3 for control, saline, and IFN-γ spleen). eAf1521 input peptides (input proteome) were analyzed using the data-dependent HCD acquisition (resolution 30 K for MS/MS) but without triggered data acquisitions. A summary of all acquisition strategies is provided in the supporting information (supplemental Fig. S3).

##### Scheduled Targeted MS of the PARP14 ADPr Peptide

A pool of mouse spleen ADPr peptides were analyzed with the same gradient as the Lumos collision energy experiments above. The PARP14 ADPr peptide (HISGLAQALSK + ADP-ribose, *m/z* 555.9058, z = 3) was analyzed using either HCD alone (CE20 or CE22%; resolution 120 K), or using HCD (CE 28% ± 3%; resolution 120 K) and EThcD on the same precursor (calibrated charge-dependent ETD parameters enabled, supplemental activation collision energy 22.5%, and resolution 120 K).

### ADP-ribosylation p-Series and m-Series Scores

The ADP-ribosylation p-series and m-series (Fig. 1*B*, supplemental Table S1) scores were calculated as follows:

$$\text{m}-\text{series}\;\text{score}=\overset{n}{\underset{k=1}{\sum }}\left(\frac{I_{k}}{I_{b}}*100\right)/n$$

$$m_{i}\;\text{ion}\;\text{score}=\frac{I_{i}}{I_{b}}*100$$

where, *k* is the index, *n* is the total number of ions considered, *i* is the ion number (1, 3, 6, and 8), *I*_*k*_ is the absolute intensity of a given *m-*ion, *I*_*b*_ is the base peak intensity.

$$\text{p}-\text{series}\;\text{score}=\overset{n}{\underset{k=1}{\sum }}\left(\frac{I_{k}}{I_{b}}*100\right)*k$$

$$p_{i}\;\text{ion}\;\text{score}=\left(\frac{I_{i}}{I_{b}}*100\right)*k$$

where, *k* is the index, *i* is the ion number (1, 3, 5, 8, and 10), *I*_*k*_ is the absolute intensity of a given *p*-ion, *I*_*b*_ is the base peak intensity.

For the m-series score, the *m1*, *m3*, *m6*, and *m8*-ions yielded the most intense signal intensities as determined from ADPr peptide spectra collected with varying HCD collision energies, thus were included for the score. For the p-series score calculation, *I*_*k*_ was always larger than $I_{k-1}$ and the individual scores were also calculated on this sorted list. The main p-series score was also optionally used as a feature for Percolator peptide validation.

### Standard MS/MS Spectral Annotation

ADPr samples' mass spectra were analyzed using a customized ADPr annotation and scoring module developed internally as an enhancement for Proteome Discoverer (PD version 2.4, Thermo Fisher Scientific). Spectral processing steps that were in common to most analyses were as follows: The spectra were queried against the Uniprot mouse (n = 63,703 entries) database (downloaded September 09, 2020) using the SEQUEST-HT algorithm. Trypsin (full) was set as the digestion enzyme, allowing up to four missed cleavages and a minimum peptide length of six amino acids. ADPr (+541.061 Da) of Asp, Glu, Lys, Arg, Ser, Thr, Tyr and His; oxidation (+15.995 Da) of methionine; and acetylation (+42.011 Da) of the N-terminus, were set as variable modifications. Carbamidomethylation (+57.021 Da) of cysteine was set as a static modification. To note, a sampled analysis of ADPr data using carbamidomethylation and ADPr of Cys as variable modifications did not yield any ADPr-cysteine modified peptides; thus, the Cys acceptor site was not considered for the remainder of the study. Spectral search tolerances were 10 ppm for the precursor mass and 20 mmu (all HCD, EThcD and CID products were measured in the Orbitrap). The peptide FDR was calculated using Percolator (target/decoy method, separate databases) and spectra were filtered based on a 1.0% or 5.0% FDR, as indicated. The “p-series score” was calculated in order to validate candidate spectra identified by SEQUEST. Subsequent XCorr thresholds for p-series scores-supported spectra were based on recommended medium confidence cutoffs in the “Fixed PSM Scorer” Node in PD (*z* = 2, 0.8; *z* = 3, 1.0; *z* ≥ 4, 1.2). Peptide-spectrum match (PSM) ranks pertain to the SEQUEST search engine rank. The proteome samples' (input peptides into ADPr workflow that were set aside) mass spectra were analyzed as above with the notable exceptions: ADPr modification was not considered, the spectra were filtered based on a 1.0% FDR cutoff, and proteins with two or more unique peptides were considered.

### Isolation Interference

Percent isolation interference was calculated using PD2.4 (details in the user manual). The calculation is used only for high resolution and accuracy scans:

$$\%\;\text{isolation}\;\text{interference}=100\times \left[1-\left(\frac{precursor\;intensity\;in\;isolation\;window}{total\;intensity\;in\;isolation\;window}\right)\right]$$

### Feature Alignment for Relative Quantification Analysis

Relative quantification was performed by the Feature Mapper and Precursor Ions Quantifier nodes. The maximum retention time shift for chromatographic alignments was set to 10 min, and the mass tolerance was set to 10 ppm. Feature linking and mapping retention time tolerance was 0, and mass tolerance was 0 ppm with a signal-to-noise threshold of 5.

### Amino Acceptor Site Analysis

EThcD scans of the *m/z* 400–1500 acquisitions (n = 6 per treatment group in the liver, for a total of 18.raw files; n = 3 per treatment group in the spleen, for a total of 9.raw files) were the input files the amino acid acceptor site profiling using the “IMP-ptmRS” node in PD2.4. High confidence search engine rank 1 ADPr peptides (Protein Group = 1) were considered. The highest probability acceptor site is reported per peptide, with a minimum probability of 95%.

### Reprocessing of Unidentified MS2 Spectra

ADPr samples' MS/MS spectra yielding “0 PSMs” (unidentified spectra) using the standard search parameters above were exported from Proteome Discoverer as.mzML files. For the HCD collision energy experiments, we considered the possibility that some of these spectra may be atypical tryptic peptides; thus we used semi-trypsin as a search parameter and decreased the number of amino acids to four. When exporting unidentified HCD spectra, only those with m-series scores ≥30 were included.

### Network Analysis

To generate protein–protein interaction networks, proteins lists were entered into the online STRING Database version 11 (21). For both liver and spleen queries, the full network and all the interaction sources (text mining, neighborhood, experiments, gene fusion databases, co-occurrences, and coexpression) were activated. Each network edge indicates a confidence level: the minimum required interaction score for the liver was set to 0.9, the highest confidence; and for the spleen it was set to 0.5, medium confidence, since the number of spleen ADPr proteins was too few for a stricter threshold. We also performed k-means clustering on the networks in order to subgroup the proteins to summarize their biological processes using the EnrichR database (22). Adjusted *p*-values provided by EnrichR were calculated by the Fisher's exact test.

### Statistical Analysis

Statistical analyses for RT-PCR data were performed using Prism (version 5.04, GraphPad Software, CA). Unpaired t-tests with Welch's correction were used to make statistical comparison between saline and IFN-γ group. Relative proteome and ADPr peptide abundances were analyzed using the statistical software, Qlucore (version 3.5, Sweden). Each analysis comparing the changes to either the proteome or ADPr peptide abundances for the liver and spleen employed a two-group comparison between saline and IFN-γ. For the liver, variances due to ADPr enrichment batches were removed from the analysis, before applying the comparison. We performed infusion, sacrifices, and organ harvest for all mice, n = 6 per group, in a single day. However, for liver samples, the ADPr enrichment was prepared in two batches (n = 3 per group per batch/separate weeks). The two-group comparisons involving liver samples therefore required an elimination of variables due to sample preparation batch effects. The three spleen samples (two pooled spleens per sample per group) were prepared at the same time; thus there were no ADPr enrichment batches to consider.

## Results

### A Complete Workflow for ADP-ribosylation Proteomics

Even though ADP-ribosylation proteomics is increasing in feasibility in the wet lab, systematic and streamlined computational methods to annotate and assess the quality of ADPr spectra do not exist. In the context of IFN-γ-induced changes to mouse liver and spleen ADP-ribosylomes, we developed a set of novel ADPr annotation scores implemented as an enhancement to Proteome Discoverer 2.4. These scores are readily incorporated into standard mass spectral annotation workflows (Fig. 1). As a source of ADPr peptides we established an acute IFN-γ-induced proinflammatory response in mice to assess the changes of the ADP-ribosylome in the liver and spleen tissues (Fig. 1*A*). In addition, we modified our ADPr peptide enrichment strategy by using a newly engineered Af1521 macrodomain (eAf1521) (19) to enrich MARylated peptides and added a molecular weight cutoff filter as an ADPr peptide cleaning step (supplemental Figs. S1 and S2). Using primarily the quadrupole ion-trap Orbitrap Fusion Lumos but also the quadrupole Orbitrap Q Exactive, we ran pilot studies using mouse liver ADPr peptides to establish the ADPr peptide annotation node. The dissociation properties of ADPr peptides have already been determined (13). The low mass “*m*-ions” are derived from the ADP-ribose and the complementary “*p*-ions” comprise the peptide plus remaining ADPr modification (Fig. 1, *B* and *C*). The corresponding *m*-ion and *p*-ion series annotations and scores (Experimental Section, ADP-Ribosylation p-Series and m-Series Scores) are implemented once candidate ADPr spectra are assigned and scored by the search engine (in this case SEQUEST-HT) (Fig. 1*D*).

The availability of the *m*-ion and *p*-ion scoring strategy permitted us to not only provide a means to evaluate and validate candidate ADPr spectra, but also to monitor and elucidate further the dissociation properties of ADPr peptides. In the following sections, we report how various instrument acquisition methods impact the number of annotated ADPr spectra. As importantly, however, we have come to understand how the corresponding *m*-ion and *p*-ion dissociation dynamics influence these annotations and how best to exploit these dynamics for future ADP-ribosylation studies.

### ADPr Spectral Confidence Reporting Is Challenged by Low XCorr Values

**Fig. 2:**
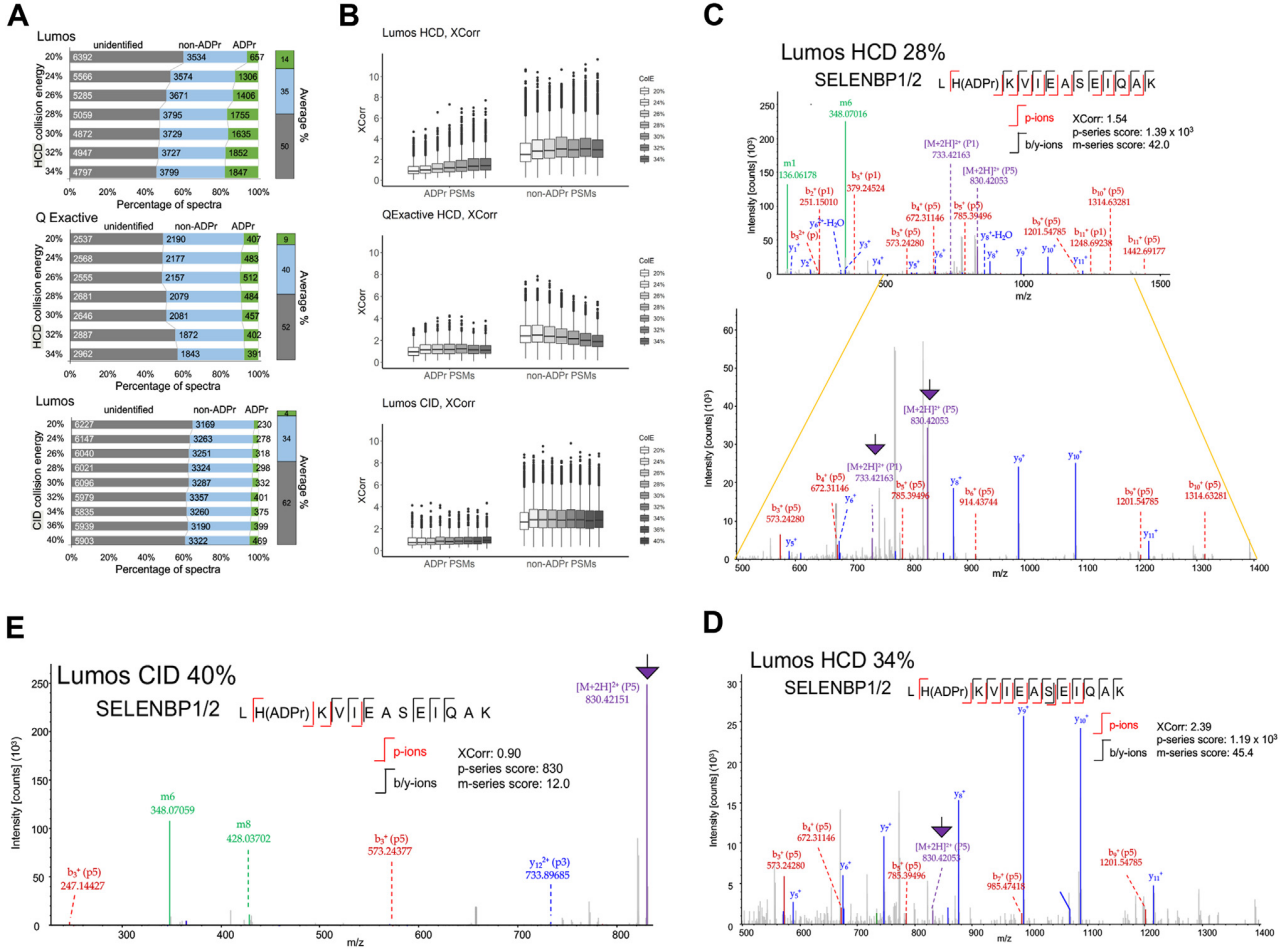
**Distributions of ADPr, non-ADPr, and unidentified spectra in collision energy experiments**. *A*, the impact of varying instrument platform, dissociation method, and collision energy on the number of annotated ADPr spectra *versus* non-ADPr and unidentified spectra (individual numbers are labeled within each bar). The average collision energy percentages are plotted to the right. Annotated spectra include only rank1 peptides (5% FDR). *B*, the relationship between XCorr and collision energy (ColE) for ADPr and non-ADPr peptides. *C*–*E*, an ADPr peptide from mouse liver Selenium-binding protein 1/2 (SELENBP1/2) sequenced by HCD CE 28% (*C*) or CE 34% (*D*) and CID CE 40% (*E*) on the Lumos. The *purple arrows* highlight precursor *P*-ions that tend to predominate at lower HCD collision energies (*e.g.*, CE 28%) or in CID spectra; but dissociate further as HCD collision energy increases (*e.g.*, CE 34%). Fully annotated spectra are in supplemental Figure S4.

Using the pilot mouse liver ADPr peptide pool, we first explored the dependency on dissociation method, collision energy, and instrument platform (supplemental Fig. S3) on the yield of ADPr PSMs. The proportion of annotated rank1 ADPr spectra (5% FDR) averaged between 4% (CID) and 14% (HCD, Lumos) and rank1 non-ADPr spectra between 34% (CID) and 40% (HCD, Q Exactive) (Fig. 2*A*). Unidentified spectra ranged between 50% (HCD, Lumos) and 62% (CID) (Fig. 2*A*). Increasing HCD collision energy on the Lumos, especially after collision energy (CE) 26%, increased the number of ADPr and non-ADPr spectra (or PSMs) (Fig. 2*A*). On the other hand, increasing collision energy on the Q Exactive increased the number of ADPr spectra up to CE 26%, but then those numbers decreased steadily until CE 34% (Fig. 2*A*). Non-ADPr spectra were relatively stable on the Q Exactive, but their numbers declined at higher collision energies (Fig. 2*A*). Increasing CID collision energy increased the number of annotated ADPr and non-ADPr steadily; however, the number of ADPr spectra peaked at only 469 (CID CE 40%) when compared with 1852 for HCD CE 32% on the Lumos (Fig. 2*A*).

Due to the predominance of *p*-ions in HCD/CID scans, XCorr values (the cross-correlation score between a spectrum and the candidate peptide) (23) are typically low for ADPr spectra (Fig. 2*B*). Increasing HCD collision energy therefore increases sequential dissociation of, from now on referred to as, precursor “*P*-ions” to fragment “*p*-ions.” A concomitant increase in XCorr with increasing HCD collision energy on the Lumos (Fig. 2*B*) suggests improved dissociation of the peptide backbone (Fig. 2*C*, supplemental Fig. S4). For example, by comparing an ADPr peptide at two HCD collision energies (Lumos CE 28% *versus* CE 34%), XCorr increases as the p-series score decreases (Fig. 2, *C* and *D*). The spectrum contains *P*-ions at collision energy 28% (Fig. 2*C*, *P1*-ion and *P5*-ion) but those *P*-ions disappeared or diminished at collision energy 34% (Fig. 2*D*), demonstrating that increasing HCD collision energy promotes *P*-ions to *p*-ion (and complementary *b*- or *y*-ions) conversion more readily. The dependence on this sequential dissociation of *P*-ion-to-*p*-ion and so on is demonstrated by the contrasting CID spectrum for the same peptide (Fig. 2*E*). In this case, the intact precursor *P5*-ion dominates the spectrum that in turn results in a low XCorr (Fig. 2*E*). Increasing HCD collision energy on the Q Exactive did not impact the ADPr XCorr values but did decrease those for non-ADPr spectra likely due to overfragmentation (Fig. 2*B*). The differences in collision energies between these two instruments have been reported (24). The collision energy offset is –6.7% for the Q Exactive relative to the Lumos (24), which is consistent with our observations that lower collision energies on the Q Exactive are optimal compared with the Lumos for identifying ADPr peptides. We present a more detailed analysis of the dependence on collision energy on the p-series' dissociation properties further below.

### The M-ion Series Score Provides Qualitative Assessment of Candidate ADPr MS2 Spectra

The ADPr peptides dissociate forming complementary *m*- and *p*-ions when using HCD or CID (Fig. 1*B*). Although they dominate MS2 spectra, the *m*-ions (m1, m3, m6, and m8) do not provide any peptide sequence information; they are nonetheless convenient to run *m*-ion-triggered MS2 strategies that increase the specificity of ADPr spectra (11). In this study, we could further exploit the diagnostic *m*-ions by using the newly implemented m-series score to evaluate the candidacy of a spectrum to be truly derived from an ADPr peptide.

**Fig. 3:**
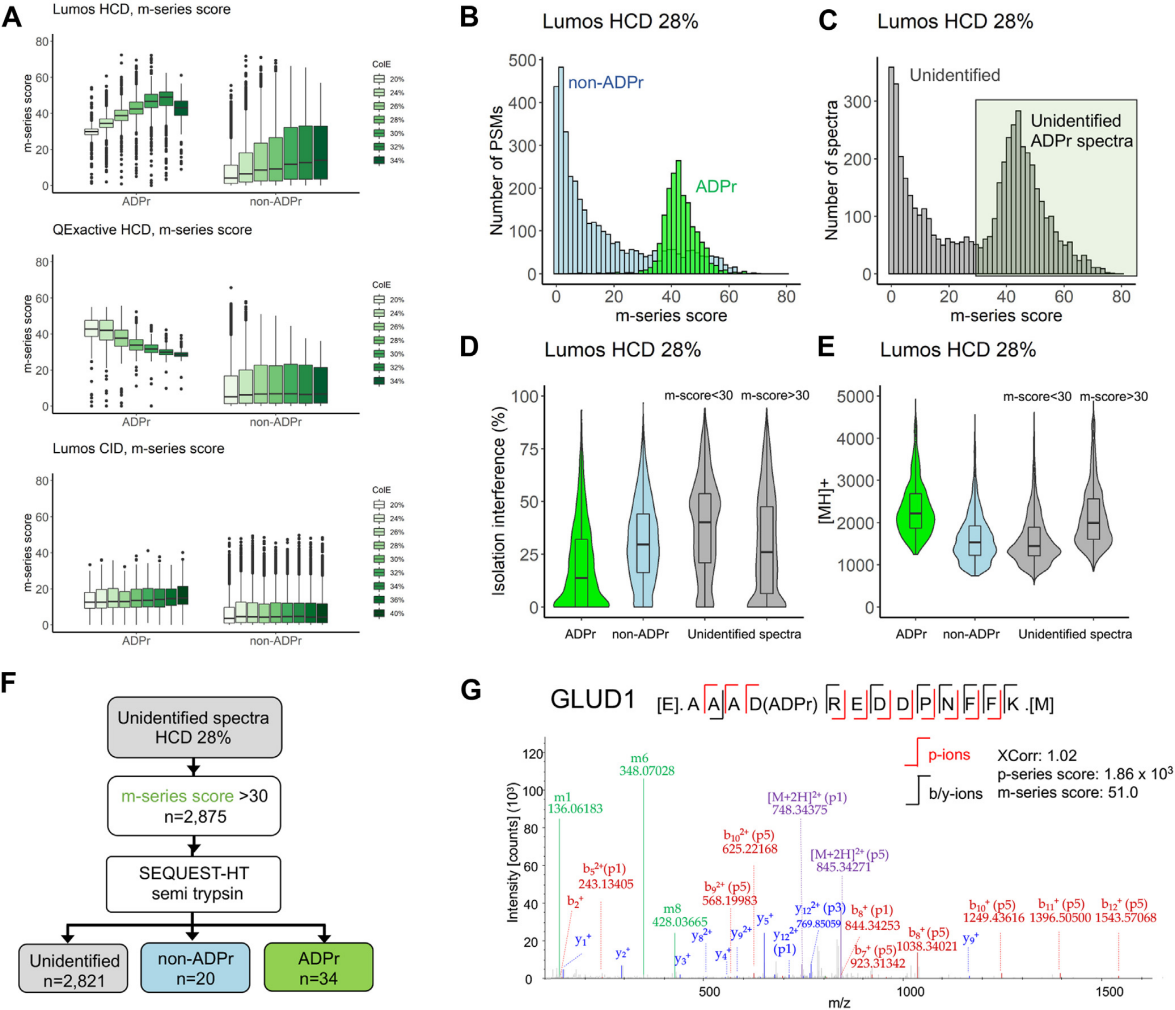
**ADPr peptide *m*-ion score distributions**. *A*, the *m*-ion series score distributions calculated from the spectra of annotated ADPr and non-ADPr PSMs (Rank 1, 5% FDR). *B*, a closer analysis of the m-series score distributions comparing Lumos HCD (28% CE)-generated ADPr and non-ADPr PSMs. *C*, the m-series score distribution for the corresponding unidentified spectra from *panel B*. Candidate ADPr spectra still contained within the unidentified spectra are highlighted. *D*, distribution of the precursor mass ([MH]+) across the spectral categories. *E*, distribution of percent isolation interference across spectral categories. *F*, an example workflow strategy (*i.e.*, semi-trypsin search) to further annotate ADPr spectra from the unidentified category. *G*, example semi-tryptic ADPr spectrum previously contained within unidentified (default fully tryptic search) spectral category.

Ideally, *m*-ions would be present only in ADPr spectra; however, coisolation of ADPr with non-ADPr peptides and potential contamination of nucleic acids (25) also results in *m*-ion contamination in non-ADPr peptide spectra. Irrespective of dissociation method and collision energy, the m-series score distributions for ADPr spectra are markedly higher and less variable (narrower interquartile ranges) than those for non-ADPr spectra (Fig. 3*A*, Rank 1 peptides, 5% FDR). Increasing the HCD collision energy on the Lumos increases the m-series score, but decreases the m-series score on the Q Exactive (Fig. 3*A*). Of note, due to the low mass cutoff in CID MS2 scans (26), the *m1*-ion is often excluded and thus the m-series scores are lower for CID data (Fig. 3*A*).

When considering all HCD collision energy conditions, a minimum m-series score of 30 is supportive of an ADPr PSM (and a score of 10 for CID spectra, Fig. 3*A*). Looking closer at the m-series distribution for one method (Lumos HCD, 28% CE), the difference in m-series score distribution across spectral classes is particularly pronounced. The m-series score for ADPr spectra peaks at 40 with the majority of ADPr PSMs populating the 30–60 score range; and the m-series score for non-ADPr spectra peaks at 5, tailing to the higher scores (Fig. 3*B*). On the other hand, unidentified spectra exhibit two peaks, consistent with a mixture of unrecognized non-ADPr and ADPr MS2 events (Fig. 3*C*). We also compared the precursor mass [MH+] distributions across the spectral classes and noted that unidentified spectra with m-series scores ≥30 have a mass distribution similar to that of ADPr spectra (Fig. 3*D*), reflective of a higher mass incurred by the ADPr modification (+541 Da). As we noted previously (11), identification of ADPr PSMs is contingent upon low isolation interference (the percentage of interference by coisolation within the precursor isolation window or the relative amount of ion current within the isolation window that is not attributed to the isolated precursor) when compared with their non-ADPr counterparts (Fig. 3*E*). These observations emphasize that if solely reliant on standard annotation workflows, ADPr spectra must be relatively interference-free to be identified with confidence. This limitation indicates that we are underreporting the true number of ADPr peptides. The m-series score can therefore be used as a diagnostic for ADPr spectral candidacy, whose spectrum is then subsequently verified by the p-series annotation (below).

### The M-ion Score Can Be Used to Further Enrich ADPr PSMs at the Spectral Processing Steps

Unidentified spectra comprise ADP peptides (Fig. 3*C*) that were not annotated for one or more reasons, including too few *b/y*-ions due to under- or overfragmentation of the peptide backbone, as confirmed by our manual inspection of these spectra. We also observed that some of these spectra harbored high-intensity fragment ions indicating that they likely comprise variables not considered in our default search. As a proof-of-concept, we extracted the unidentified spectra (Lumos, HCD CE 28%) with an m-series score ≥30 (n = 2875) and reprocessed them using semi-trypsin as a search parameter (Experimental Section, Fig. 3*F*). We retrieved an additional 34 annotated ADPr PSMs (Rank 1, 5% FDR) and 30 non-ADPr PSMs, with the rest remaining as unidentified (Fig. 3*F*). An example semi-tryptic ADPr spectrum is fully annotated when considering both *b/y*-ions (XCorr = 1.02) and fragment *p*-ions (p-series score = 1860) (Fig. 3*G*).

### ADPr Peptides' Dissociation Dynamics Differ Across Acquisition Methods

The *m*-ion series score changes with increasing HCD collision energy (Fig. 3*A*) implying that the complementary *P*-ion and *p*-ion intensities are also changing, which in turn could impact the dependence on the *p*-series score to validate ADPr peptide spectra. We thus examined the individual *m*-ion intensities generated by increasing HCD collision energy on the Lumos and Q Exactive with the aim to monitor the trends of their complementary *P*-ions.

Firstly, we noted the following striking features of the *m*-ion series: the *m1*-and *m6*-ion signals dominate the m-series score; and the *m1*-ion intensity increases, whereas the *m6*-ion intensity decreases with increasing HCD collision energy (supplemental Fig. S5*A*). In contrast, the predominant *m6*-ion and the low abundant *m8*-ion are relatively stable with increasing CID collision energy (supplemental Fig. S5*A*). Secondly, we examined the complementary p-series score distributions by monitoring the total p-series score and its breakdown into the precursor *P*-ion and fragment *p*-ion scores (supplemental Fig. S5*B*). We also generated a further breakdown of the *P*-ion series into the individual *P1*-ion, *P3*-ion, *P5*-ion, *P8*-ion, and *P10*-ion scores (supplemental Fig. S6), to directly monitor their relationships with their complementary *m*-ions. The p-series and the *P*-ion scores decrease with increasing HCD collision energy for both the Lumos and Q Exactive but remain stable with CID (supplemental Fig. S5*B*). Specifically, the *P5*-ion dominates HCD and CID MS2 spectra, but there is a shift toward the *P1*-ion (complete loss of the ADPr moiety) as HCD collision energy increases (supplemental Fig. S6).

The *p*-ion scores, on the other hand, exhibit diverse trends across the three collision energy conditions—they remain stable with HCD on the Lumos, decreasing with HCD on the Q Exactive, and increasing slightly with CID on the Lumos (supplemental Fig. S5*B*). The differences in the *p*-ion score distributions indicate that depending on the instrument platform and collision energy, the reliance on *p*-ion series annotations to validate ADPr MS2 spectra will vary. For instance, in order to leverage both XCorr and the *p*-ion scores, HCD on the Lumos can be performed at collision energies ≥26% and from 20% to 26% on the Q Exactive (supplemental Fig. S5*C*). Although CID is not routinely used for ADP-ribosylation studies, higher collision energies promote both increased XCorr and overall p-series scores (supplemental Fig. S5*C*).

### The ADPr m1-ion Forms Primarily from the Dissociation of the Larger m-ions

**Fig. 4:**
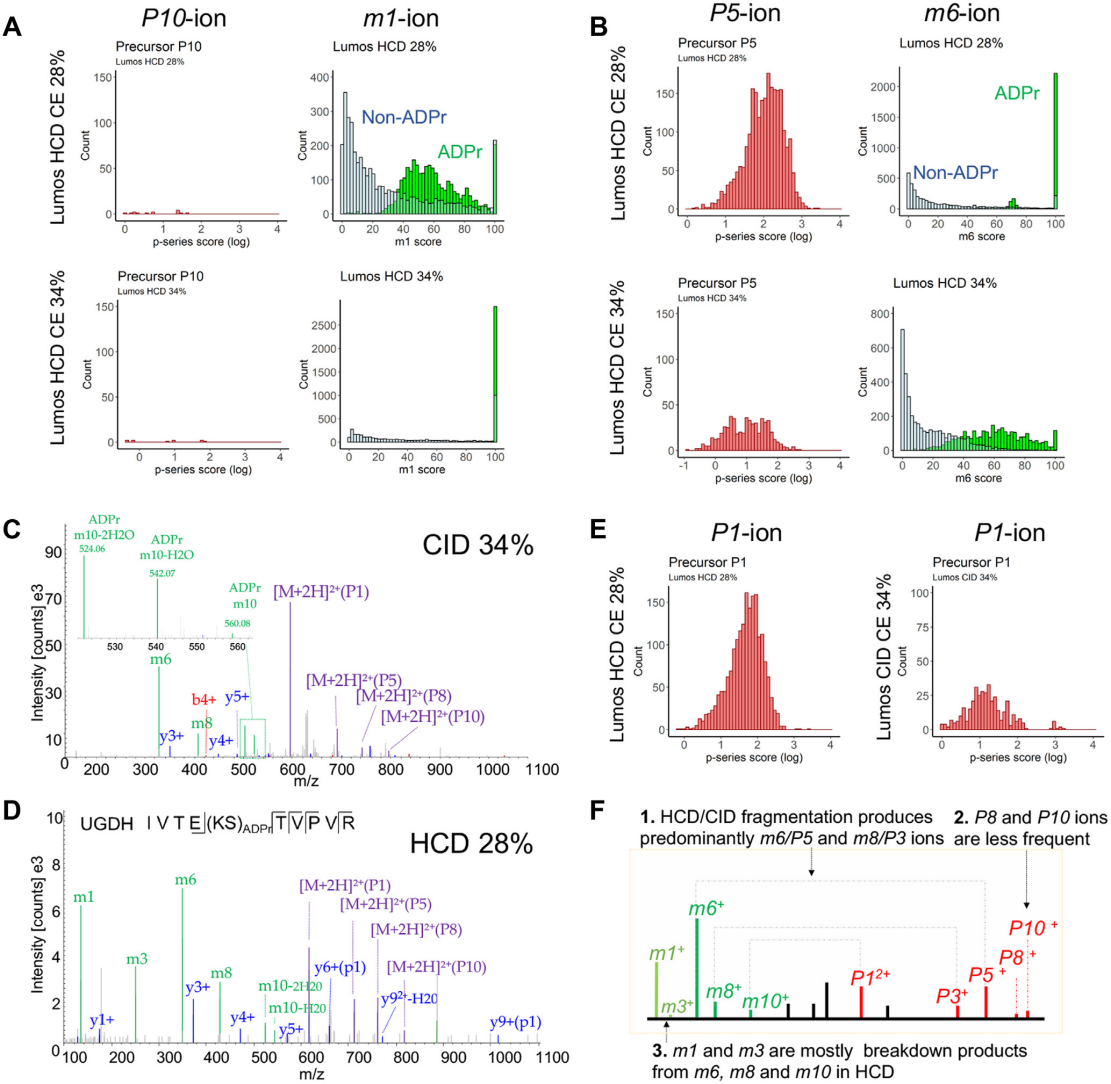
**The *m*-ions undergo sequential dissociation**. *A*, the *m1*/*P10*-ion dynamics with two HCD collision energy settings on the Lumos. *B*, the *m6*/*P5*-ion dynamics with two HCD collision energy settings on the Lumos. *C* and *D*, an ADPr peptide analyzed by CID (*C*) or HCD (*D*) on the Lumos, demonstrating that the ADPr molecule is more readily detected in CID but not HCD, likely due to sequential dissociation. *E*, the *P1*-ion is prevalent in HCD and CID scans, despite the low signal for the corresponding *m10*-ion in HCD data, suggesting the *m10*-ion dissociates further in HCD. *F*, A summary of the dissociation properties of ADPr peptides when using HCD.

An additional observation from monitoring the formation of *m*-ions and *p*-ions over collision energies is that the predominant *m1*-ion and lower abundant *m3*-ion in HCD data are formed due to continued dissociation of the *m6*-and *m8*-ions (supplemental Fig. S5*D*), rather than solely due to the direct dissociation of the ADPr peptide to form the *m1/P10*-ion or *m3/P8*-ion pairs. For instance, CID spectra maintain stable m6-ion and *m8*-ion signals with increasing collision energy, without any notable increase in the *m3*-ion signal (the *m1*-ion cannot be readily captured due to the low mass cutoff rule) (supplemental Fig. S5*D*). Direct infusion of AMP (*m6*-ion) demonstrates this *m6*-to *m1*-ion conversion rather handedly (supplemental Fig. S5*E*). In addition, the *m1*-ion score increases even though higher collision energies do not increase the prevalence of the complementary *P10*-ion (Fig. 4*A*). On the other hand, the *m6*-ion score decreases as its complementary *P5*-ion decreases with increasing collision energy (Fig. 4*B*). Moreover, we cannot rule out that the *m10*-ion (intact ADPr moiety), although very low in abundance in HCD scans and not considered in the m-series score, is a source of the lower mass *m*-ions. We noted that in CID spectra, the *m10*-ion is more readily detected (Fig. 4*C*), compared with the peptide's HCD counterpart (Fig. 4*D*); yet, the complementary *P1*-ion is still prominent in HCD data (Fig. 4*E*). We therefore interpret these data to indicate that with HCD, once the complete ADPr modification is lost, it continues to dissociate, and contributing to one or more of the lower mass *m*-ions (Fig. 4*F*). Taken together all observations to this point, for the majority of ADPr peptide spectra, validation *via* the p-series score will most likely be dependent on the precursor/fragment *P1*-/*p1*-, *P3*-/*p3*-, and *P5*-/*p5*-ions. In addition, the *p3*- and *p5*-ions (and *p8*- and *p10*-ions if observed) will facilitate amino acid localization since they retain part of the ADPr moiety.

### P-series Score Facilitates Inclusion of Low Scoring Spectra as ADP-ribosylated

**Fig. 5:**
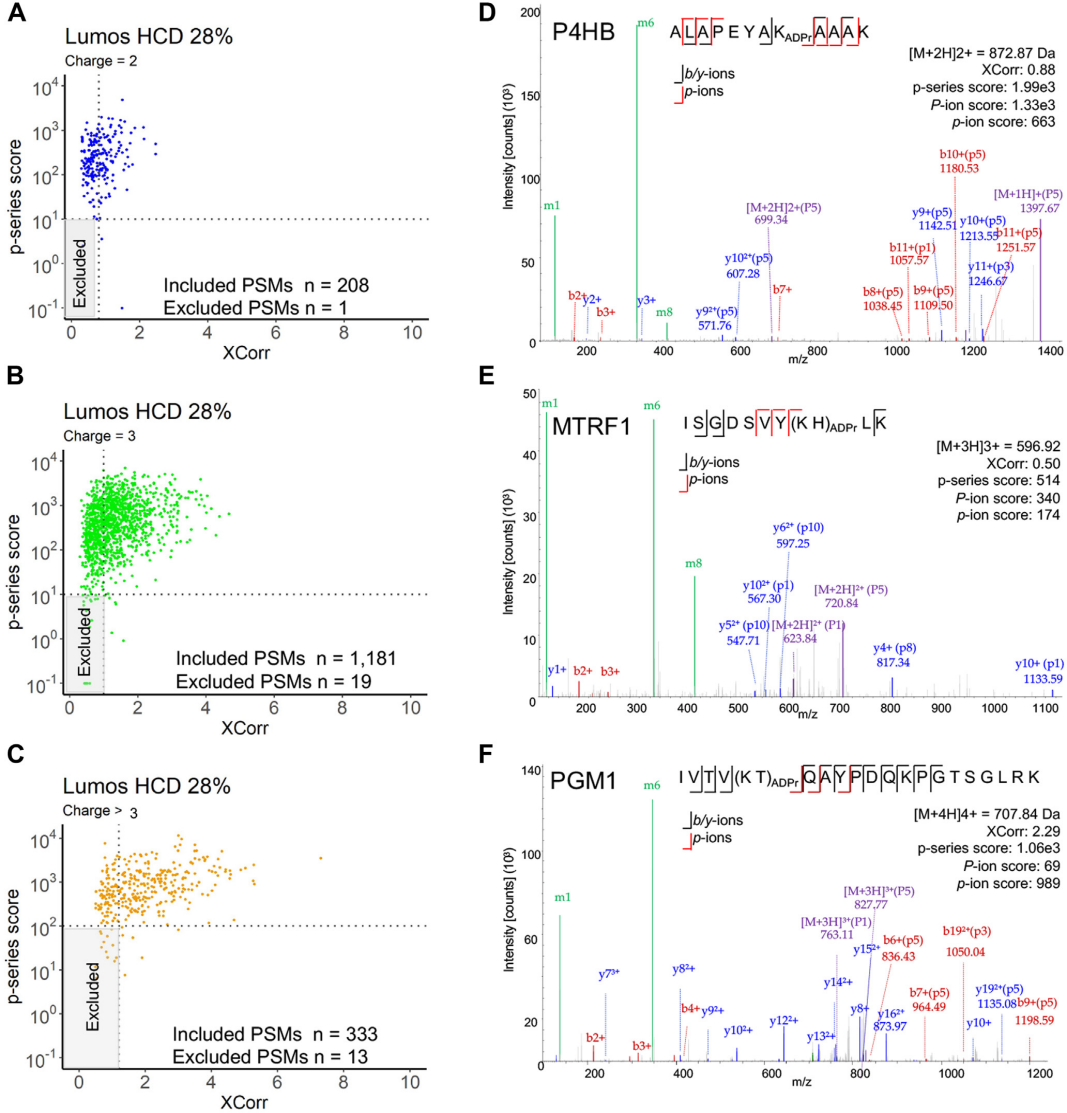
**P-series support inclusion of medium confidence ADPr peptide identifications**. *A*–*C*, p-series *versus* XCorr grouped by precursor charge. PSMs are rank 1, 5% FDR. XCorr thresholds (0.8, *z* = 2+; 1.0, *z* = 3+; 1.2, *z* > 3+) are based on the Fixed PSM Scorer's medium confidence thresholds. The p-series cut-off was based on manual inspection of several spectra. PSMs inside of gray area will not be considered further. *D*–*F*, example medium confidence (5% FDR), low XCorr ADPr spectra supported by p-series ions. MTRF1, Peptide chain release factor 1; P4HB, Protein disulfide-isomerase; PGM1, Phosphoglucomutase-1.

The final evaluation of the ADPr peptide annotation workflow using the pilot liver data was to determine whether the p-series score could be used to systematically evaluate and increase the number of reportable ADPr peptides. Candidate ADPr peptide spectra may rely on one or both p-series ions for validation; however, lower scoring spectra supported by *p*-ions are more beneficial since they provide peptide sequence information and the potential to localize the amino acid acceptor site. We examined more closely the HCD (*i.e.*, 28% CE) data from the Lumos and noted that as the ADPr peptides' charge states increase, the higher the p-series score cutoff required to include lower XCorr spectra (cutoff based on the Fixed PSM Score in Proteome Discoverer) (Fig. 5, *A*–*C*, supplemental Fig. S7*A*). For example, precursor charges 2+ and 3+ benefit from a p-series score cutoff of 10 (Fig. 5, *A* and *B*), but higher charge states ≥4+ that comprise larger peptides (supplemental Fig. S7*B*) require a more stringent cutoff of 100 (Fig. 5*C*). Example lower XCorr spectra from protein disulfide-isomerase (P4HB), mitochondrial fission regulator 1 (MTFR1), and phosphoglucomutase (PGM1) demonstrate that the *p*-ions corroborate the sequence identification (Fig. 5, *D*–*F*).

### HCD and EThcD Analysis of Mouse Liver and Spleen ADP-ribosylomes

We established an *in vivo* model for acute proinflammatory responses using an intraperitoneal injection of IFN-γ. Each organ contains cells that are known responders to IFN-γ: the spleen is a reservoir for monocytes and other immune cells (27) and the human liver cell line, HepG2, is known to exhibit a typical IFN-γ response (28). Moreover, *PARP14* mRNA is highly expressed in human spleen as documented on proteinatlas.org (supplemental Fig. S8*A*).

**Fig. 6:**
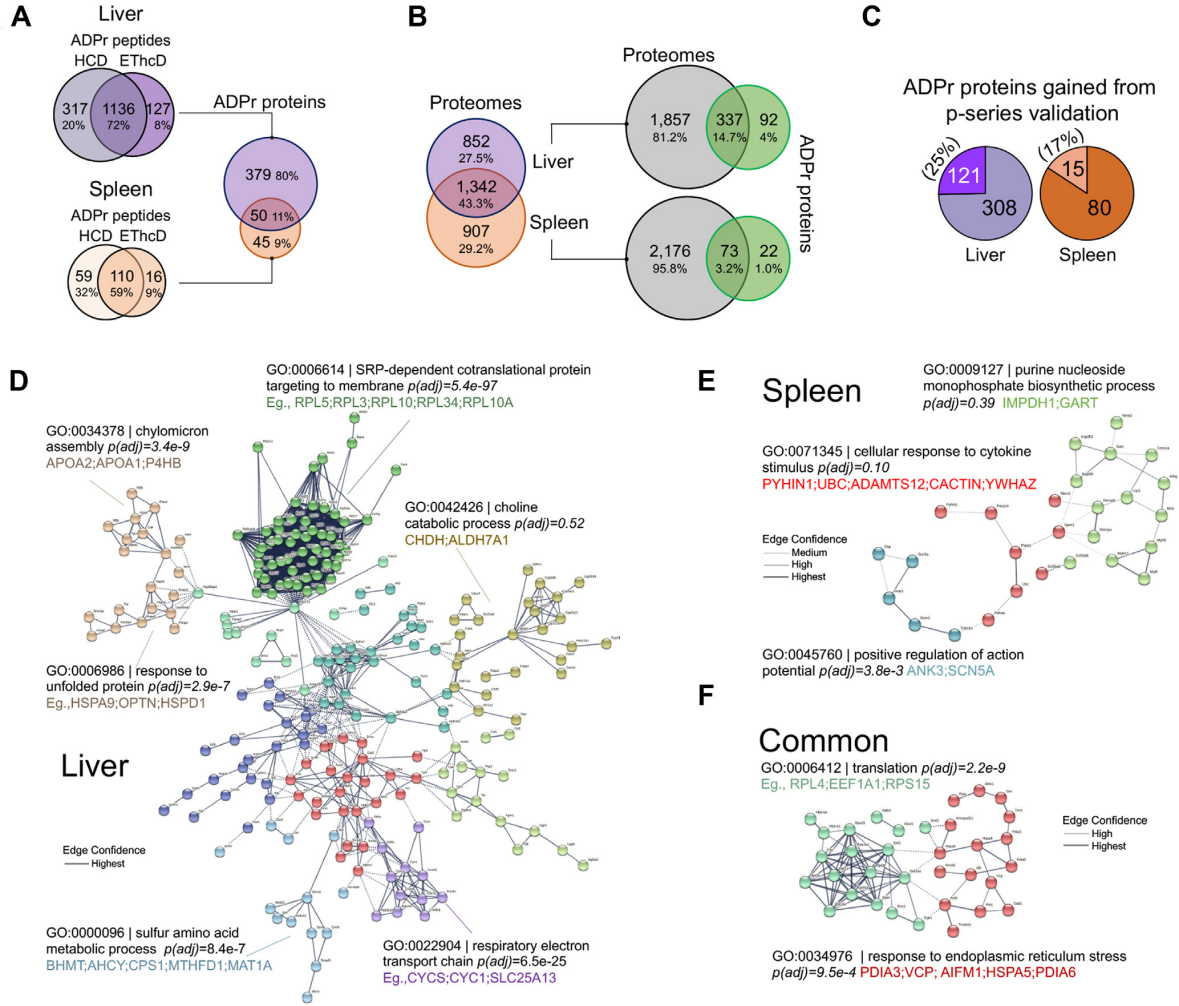
**The distinct ADP-ribosylomes from mouse liver and spleen**. *A*, total Rank 1 unique ADPr peptide sequences identified using HCD and EThcD events triggered from the same precursor scan, and their corresponding proteins. *B*, overlap between liver and spleen proteomes and liver and spleen ADPr proteins with their respective proteomes. The proteomes were analyzed with a single HCD acquisition, whereas the ADP-ribosylomes were analyzed by multiple gas-phase separation (GPS) acquisitions. Proteins from each portion of the Venn diagram are in supplemental Table S4. *C*, total ADPr proteins reported. *D*, STRING database “full network” output for the liver unique ADPr proteins: 361/379 proteins in the database; highest confidence edge strength reported, 0.900. *E*, STRING database “full network” output for the spleen unique ADPr proteins: 43/45 proteins in the database; at least medium confidence edge strength reported, 0.400. *F*, STRING database “full network” output for the overlapping common ADPr proteins: 47/50 in the database; at least high confidence edge strength reported, 0.700. For *C–E*, disconnected nodes are hidden; and k-means clusters (n = 10 for liver; n = 3 for spleen; n = 2 for common proteins) were chosen manually after iterations of varying cluster number with subsequent Gene Ontology analysis. *Dashed lines* are interactions separated by clusters.

We confirmed that the liver and spleen organs were responsive to IFN-γ as gauged by the increase in mRNA levels of IFN-γ-inducible chemokines (*e.g.*, *Ccl2*) and *Parp14* and *Parp9* (11, 18) (supplemental Fig. S8, *B* and *C*). On the other hand, *Parp1* mRNA tended to decrease, but this decrease was not significant (supplemental Fig. S8, *B* and *C*). Before analyzing IFN-γ-induced changes to each organ, we combined ADPr peptide data from each condition, control, saline, and IFN-γ and compared the ADPr peptide and protein profiles between the liver and spleen (supplemental Tables S2 and S3). EThcD and HCD ADPr peptide identifications overlap well, 72% and 59% for the liver and spleen, respectively (Fig. 6*A*). These ADPr peptides corresponded to 429 liver and 95 spleen proteins of which 50 (11%) overlapped (Fig. 6*A*). In contrast, the overlap of common proteins detected in the liver and spleen proteomes is 43.3% (Fig. 6*B*), most likely due to the equal depth in proteome sequencing in the latter. With a relatively limited number of spleen ADPr proteins compared with those from liver, interorgan comparisons are not informative. The majority of ADPr proteins were identified in each organ's proteome, whereas 92 and 22 ADPr proteins were not accounted for in the liver and spleen proteomes, respectively (Fig. 6*B*, supplemental Table S4). In addition, we gained 25% (liver) and 17% (spleen) additional ADPr proteins by implementing the p-series annotation and score (Fig. 6*C*).

Liver-unique ADPr proteins (from Fig. 6*A*) form networks related to SRP-dependent cotranslational protein targeting to membrane (green cluster), protein folding and lipoprotein assembly (gold cluster), metabolism and catabolism (light blue and olive-green clusters), and respiratory electron transport chain function (purple cluster) (Fig. 6*D*). However, spleen-unique ADPr proteins (from Fig. 6*A*) are associated with purine nucleoside monophosphate synthesis (green cluster), cytokine response (red cluster), and regulation of action potential (blue cluster) (Fig. 6*E*). ADPr proteins common to the liver and spleen are primarily related to protein translation (Fig. 6*F*).

### Mouse Liver and Spleen Respond Differently to Systemic IFN-γ

**Fig. 7:**
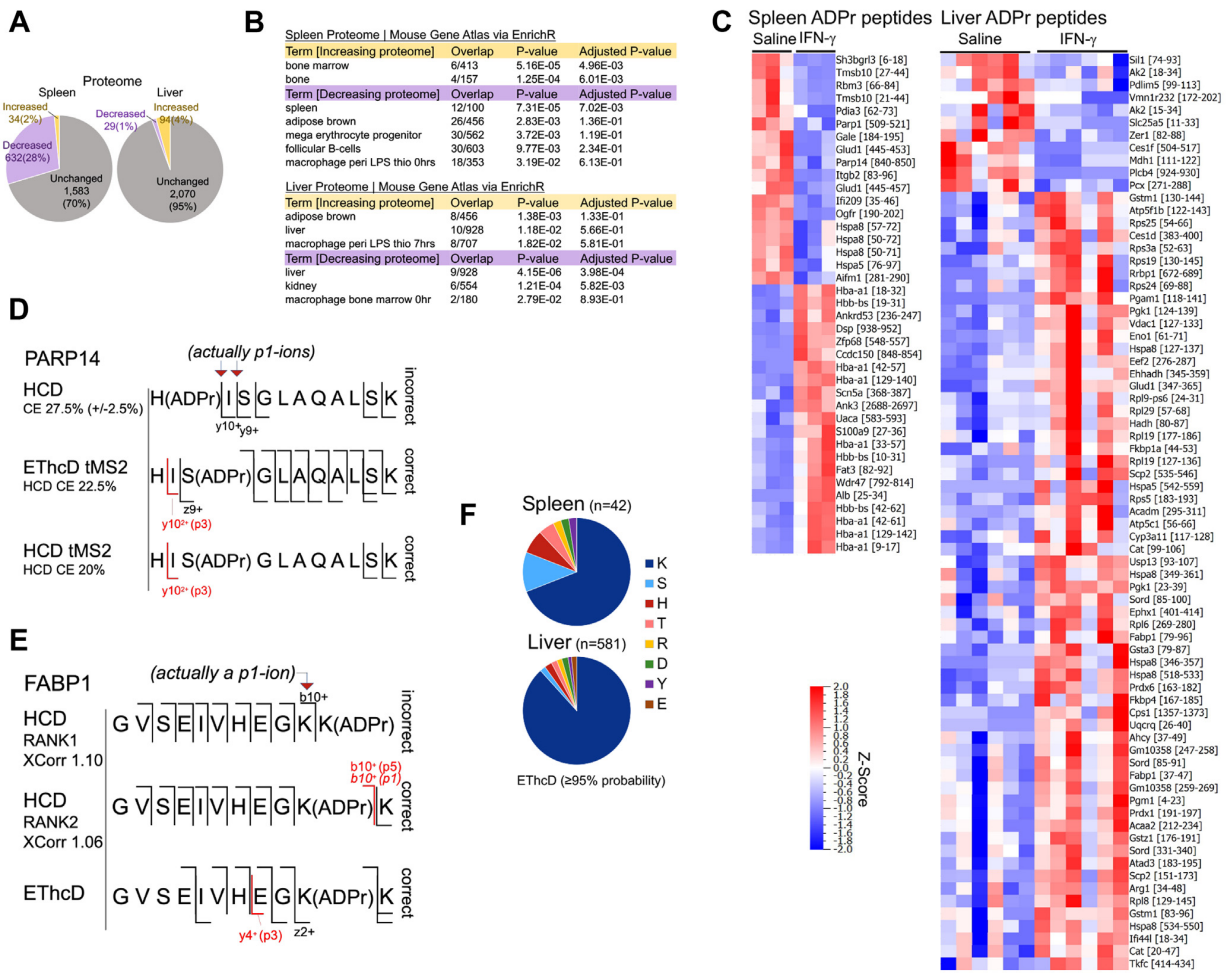
**IFN-γ induces distinct responses in mouse liver and spleen**. *A*, changes to mice spleen and liver proteomes in response to IFN-γ. The average of n = 3 (spleen pools) and n = 6 (liver) for saline *versus* IFN-γ. *B*, cell and tissue-specific terms recognized by the Mouse Gene Atlas database for IFN-γ-induced proteome changes. *C*, heat maps of two-group comparisons of changing ADPr peptides (*p* < 0.05; q = 0.23 spleen and q = 0.62 liver). [Gene name and [amino acid position]. *D*, *p1*-ions are assumed to be *y*-ions by the search engine. Target MS2 (tMS2) of a candidate His-modified ADPr confirms the downstream Ser to be the correct site. *E*, a *p1*-ion is assumed to be a *b*-ion by the search engine. The p-series annotation indicates the lower ranking (rank 2) peptide to be correct; EthcD supports the rank2 assignment. *F*, EThcD-based summary of ADPr acceptor sites. Only the GPS scan *m/z* 400–1500 was used for the acceptor site consensus. The highest scoring amino acid site for a given peptide sequence is reported.

Spleen and liver proteomes exhibited contrasting responses to IFN-γ. A markedly large proportion of the spleen proteome decreased (28%), whereas only 2% increased in abundance (Fig. 7*A*). Liver on the other hand exhibited a more balanced response with a 4% increase and 1% decrease in the quantified proteome in response to IFN-γ (Fig. 7*A*). The Mouse Gene Atlas database analysis of these proteins indeed recognized blood and immune cell types within the top five outputs for decreased spleen proteins (mega erythrocyte progenitor, follicular B-cells, and macrophages) (Fig. 7*B*). On the other hand, while macrophages were indicated in both increased and decreased liver proteins, the term was supported by fewer protein overlaps compared with spleen (Fig. 7*B*). Proteins that increased in the liver and decreased in the spleen were also associated with adipose tissues (Fig. 7*B*), primarily due to metabolic term such as fatty acid biosynthesis, owing to enzymes such as acetyl-CoA carboxylase 2 (ACAB2 in the liver), and medium-chain specific acyl-CoA dehydrogenase and small-chain specific acyl-CoA dehydrogenase (ACADM and ACADS, respectively in the spleen).

Cytokine responsive genes whose proteins increased in the liver included “signal transducer and activator of transcription” members STAT1 and STAT3 and “interferon gamma-induced GTPase” IGTB (supplemental Table S5). In the spleen on the other hand, STAT2, STAT3, and STAT5a were all decreased (supplemental Table S6). Of note, we detected PARP enzymes only in the spleen. PARP1 and PARP3 decreased, whereas PARP9 increased in response to IFN-γ (supplemental Table S6), consistent with the *Parp1* and *Parp9* mRNA data (supplemental Fig. S8*C*).

### Changing ADPr Peptides Represent a Complex Tissue Response to IFN-γ

ADP-ribosylation, based on Western blot analysis, demonstrated that the net ADP-ribosylation signal in the liver, although variable, remained relatively stable up to 8 h post-IFN-γ injection when compared with the dramatic decrease in the spleen (supplemental Fig. S9). These observations, in addition to the decrease in the proteome (Fig. 7*A*), suggest that immune cells likely vacated the spleen and entered circulation in response to IFN-γ (29). Rather interestingly, the overall changes to the ADPr peptides from the liver and spleen do not reflect the anti-ADPr Western blot analysis. Using a two-group comparison (saline *versus* IFN-γ; *p* < 0.05; q = 0.62 liver, q = 0.23 spleen), the majority of liver ADPr peptides decreased, whereas the numbers of increased and decreased spleen ADPr peptides were similar (Fig. 7*C*). In particular, several spleen ADPr peptides that increased are from hemoglobins HBA and HBB; however, these proteins' abundances also increased (supplemental Table S7). We therefore cannot discern whether the increases in these ADPr signatures are due to an increase in the baseline protein or to an increase in ADPr status itself. On the other hand, most proteins corresponding to altered liver ADPr peptides (increased or decreased) did not change in response to IFN-γ (supplemental Table S8). Specifically, two “fatty acid binding protein 1” FABP1 ADPr peptides increased, whereas FABP1 itself remained unchanged with IFN-γ (Fig. 7*C*, supplemental Table S8).

### The P-ion Series Can Facilitate Amino Acid Acceptor Site Validation

HCD scans provided us more ADPr peptide identifications when compared with EThcD (Fig. 6*A*), but EThcD is superior for amino acid acceptor site localization (11, 20). In particular, we described a PARP14 ADPr peptide (HISGLAQALSK) as decreasing in the spleen IFN-γ treated group (Fig. 7*C*). This peptide was originally identified only by HCD and was annotated by the search engine as being modified at the n-terminal histidine (Fig. 7*D*). The presence of unmodified *y*-ions immediately downstream of the histidine (*y10* and *y9*) supported the histidine as the acceptor site (Fig. 7*D*, supplemental Fig. 10*A*). However, with the understanding that *p1*-ions are indistinguishable from unmodified fragment ions, we performed targeted MS2 (tMS2) with EThcD (HCD CE 22.5%). tMS2 confirmed our notion that a downstream amino acid, in this case the serine-3, is the correct acceptor site, as supported by the targeted EThcD analysis (Fig. 7*D*, supplemental Fig. 10*B*). Moreover, tMS2 at HCD collision energy 20%, although not ideal for backbone fragmentation, provided support for the serine-3 by the preservation of the *y10+ p3*-ion (Fig. 7*D*, supplemental Fig. 10*C*). In a second example, we examined more closely the changing liver FABP1 ADPr peptide (GVSEIVHEGKK that increased with IFN-γ) whose search engine rank1 assigned the c-terminal lysine as ADP-ribosylated (Fig. 7*E*, supplemental Fig. 11*A*). Again, the assignment was supported by the presence of the presumed *b10*-ion that instead, is annotated as a *p1*-ion, along with the definitive *p3*-ion, when the lysine-10 is assigned as ADP-ribosylated for the rank2 peptide (Fig. 7*E*, supplemental Fig. 11*B*). The parallel EThcD scan (same precursor ion) also supports the ADPr assignment to the lysine-10 (Fig. 7*E*, supplemental Fig. 11*C*).

Despite these specific examples we have not yet explored methods that can evaluate acceptor sites using the p-series at a wide-scale level. We therefore relied on available EThcD scans whose amino acid assignment probabilities were ≥95% to summarize the acceptor site trends. When considering the combined data for all three treatment groups (control, saline, INF-γ) for each organ (see Experimental Section), the predominant acceptor site is lysine for both the spleen and liver (Fig. 7*F*, supplemental Table S9). Although we sequenced far fewer ADPr peptides in the spleen, serine and histidine are the second most abundant acceptor sites, whereas all other acceptor sites are more evenly distributed for the liver (Fig. 7*F*).

## Discussion

ADP-ribosylome studies are increasing, but those that are reliant on HCD face numerous challenges. The ADPr moiety is unstable and inconsistently so depending on collision energy and instrument platform as we have demonstrated and depending on the amino acceptor site (15, 30). The resulting fragment spectra therefore contain signals owing to backbone *b/y* ions and neutral or modifications loss ions (resulting in the precursor “*P*-ions”), but also to dissociation of both the peptide and ADPr bonds (fragment “*p*-ions”) that are not considered during annotation. Due to the prevalence of *P*-ions and *p*-ions at the cost of HCD-generate *b*/*y*-ions, ADPr peptide spectral scores (*i.e.*, XCorr) are lower than those of unmodified peptides and are less likely to pass spectral quality criteria. ETD-dependent strategies provide one solution to ADPr lability since they shift fragmentation to favor the peptide backbone's dissociation (17); however, HCD-dependent peptide sequencing is more widely used; thus computational strategies that can address spectral quality are warranted. We therefore implemented an ADPr peptide annotation module that is inserted post-spectral searching and scoring. This node provides measures of ADPr peptide spectral candidacy, based on the *m*-ion series score, and ADPr peptide validation based on the *p*-series score, with the latter supporting lowering spectral score thresholds, thereby increasing the number of reportable ADPr peptides and proteins.

Before investigating the effects of IFN-γ infusion on mice spleen and liver, we used a pilot study's liver ADPr peptide samples to perform collision energy experiments that demonstrated the following ADPr peptide fragmentation properties observed on the Lumos and Q Exactive: (1) The *P5*-, *P3*-, and *P1*-ions are the predominant *P*-ions; but only the corresponding *m6*-ion, and not the *m8*-and *m10*-ions, respectively, prevails in HCD scans. (2) The *m1*-ion, although prevalent in HCD spectra, is formed primarily from the dissociation of the larger *m*-ions, with some contributions from the formation of the complementary *P10*-ion. (3) When considering the *m1*-, *m3*-, *m6*-, and *m8*-ions, an m-series score ≥30 is diagnostic of a spectrum containing an ADPr modification for spectra solely dependent on HCD, provided that the spectra are validated with subsequent annotations including the p-series annotation. (4) *P*-ions dissociate into *p*-ions with increasing collision energy (supplemental Fig. S5). (5) The p-series score distribution varies markedly with instrument platform and collision energy. (6) The p-series score can be used to justify inclusion of lower confidence ADPr peptides. (7) The m-series and p-series scores are useful metrics to evaluate further the properties of HCD-generated ADPr peptide spectra.

Since the *m*-ions are diagnostic for the presence of an ADPr moiety and are not unique to the peptide itself, the m-series score can be calculated for all MS2 spectra. The annotation of *P*-ions and *p*-ions and calculation of the p-series score, however, are contingent upon the search algorithm rendering a spectrum an ADPr peptide candidate. Unannotated MS2 spectra that may be plentiful with ADPr peptides can be thus be inferred from the m-series scores. This systematic annotation and scoring of the *m*-ion and *p*-ion series ions will therefore facilitate studies aiming to characterize basal levels or mild stimulants such as IFN-γ induction of PARP enzyme activities, whose ADPr peptide yields are less than those recovered from the potent stimulant H_2_O_2_ (17).

In this study, we confirmed that the liver and spleen responded to IFN-γ injection by the increase in mRNA of prototypical IFN-γ-responsive genes including *Parp14 and Parp9*. Quantitative analysis of the proteomes indicated that the spleen was more responsive, based on the marked reduction of proteins in the IFN-γ group. The dramatic decrease in the spleen proteome may be due to a mass release of monocytes that are known to vacate the subcapsular red cap in response to acutely or chronically damaged tissues such as an infarcted myocardium or atherosclerotic plaques, respectively (27, 29, 31). On the other hand, there still remain resident macrophages and dendritic cells in the marginal zone (29) that could have contributed to the increase in mRNA of the IFN-γ responsive genes. The liver's response on the other hand was more balanced for increasing and decreasing proteins. The liver comprises several cell types including hepatocytes, Kupffer cells, sinusoidal endothelial cells, and fat storing cells (32), any of which could have responded to IFN-γ, but unlike the spleen do not evacuate the organ upon a stress.

The observed changes to the liver and spleen ADP-ribosylomes are more challenging to interpret. The anti-ADP-ribosylation Western analysis indicated a dramatic decrease in spleen ADPr signal up to 8 h post-IFN-γ, whereas that from liver was variable over the same time course, yet relatively stable when compared with the spleen's response. On the other hand, quantitative analysis of the ADPr peptides resulted in a net increase in ADPr peptide signal in liver and a balance in the increasing and decreasing responses in spleen. The differences between Western blot and quantitative ADPr peptide analysis are not contradictory per se but emphasize that the former is too vague and the latter too specifically a descriptor of ADPr signaling events. For instance, a decrease in an ADPr peptide's abundance can be due to a removal of the ADPr modification itself, but also to a net decrease in the total protein abundance, a hyper-ADP-ribosylation event on the surrounding amino acids, or the addition of another posttranslational modification. Thus, a loss in a modified peptide's abundance is not exclusive to a reduction in the PTM's occupancy (33); and vice versa, an increase could result as a result of the opposite events described above. Moreover, the complexity of cell types comprising each organ makes it difficult to determine the cellular source(s) of the final ADPr signatures. As ADP-ribosylome workflows improve, however (17), there will be lesser reliance on milligrams protein inputs to enrich ADPr peptides. Studies aiming to sort cells from complex tissues that in turn reduce protein yields will inevitably be possible, in a manner similar to the evolution of phosphoproteomics workflows (34).

We previously studied the IFN-γ-induced proteome and ADP-ribosylome changes to the human macrophage-like cell line, THP-1 (11), that when compared with the *in vivo* experiments yielded more consistent ADP ribosylation trends. In THP-1 cells, the anti-ADP-ribosylation Western signal increased steadily up to 12 h and the corresponding changes to ADPr peptides also skewed toward increased ADPr peptide abundances (11). Proteins whose ADP-ribosylation statuses increased in THP-1 cells included PARP9 and PARP14, and several ribosomal subunits associated with SRP-dependent protein translation; whereas those that decreased were relatively few, including PARP1 (8, 11). In particular, we previously observed an increase in ADPr peptide abundances for both PARP9 and PARP14 that were independent of the increase in the protein abundances (11). In this current study, we identified a distinct spleen PARP14 ADPr peptide that decreased in response to IFN-γ. Although we did not detect PARP14 in the proteome, we expect that the increase in its mRNA supports an increase in PARP14 protein, since PARP9 protein was detected and increased in IFN-γ.

Despite the identification of multiple ADPr sites on PARP14 described previously (10, 11, 17), the functional roles of these ADPr sites are not known. The PARP14 ADPr peptide identified in the spleen (840_HIS(ADPr)GLAQALSK_850), however, underscores the challenges associated with HCD-dependent acceptor site mapping of posttranslational modifications (15, 30). If solely dependent on HCD, the n-terminal histidine would have been assigned as the acceptor site, but EThcD confirmed the downstream serine to be the true acceptor site. Moreover, as guided by the observations that a lower collision energy on the Lumos shifts away from complete loss of the ADPr (*p1*-ion) and toward *p*-ions retaining residual ADPr (*i.e.*, *p3*-ion in Fig. 7*D*, supplemental Fig. S6), we could demonstrate the serine to be the correct acceptor with HCD. Similarly, the *p5*-ion for FABP2 ADPr peptide supported the search engine rank2 (lysine-10) assignment over the rank1 (lysine-11) (Fig. 7*E*), underscoring the practicality of employing the *p*-ion series annotation. It is also imperative to note that for each case, we detected only a single chromatographic peak for each PARP14 and FABP1 ADPr peptide, indicating that our analysis was not complicated by overlapping ADPr peptide forms.

In summary, our study presents initial and critical steps toward advancing ADPr peptide spectral annotation. The workflow improves HCD-generated ADPr peptide confidence by incorporating the *p*-ion series annotations and scores. As interestingly, both *m*-ion and *p*-ion series scores can guide researchers through alternative mass spectrometric acquisition methods and data processing strategies, which in turn improve sequencing depth, with the potential to increase the breadth of biological findings.

## Data availability

The mass spectrometry and resulting search data have been deposited to the ProteomeXchange Consortium *via* the PRIDE (35) partner repository with the dataset identifier PXD027454 and 10.6019/PXD027454.

## Supplemental data

This article contains supplemental data (19).

## Conflict of interest

B. D. and W. N. are employees of Thermo Fisher Scientific. All other authors declare no competing interests.
