# Supplementary material for: A Novel Spectral Annotation Strategy Streamlines Reporting of Mono-ADP-ribosylated Peptides Derived from Mouse Liver and Spleen in Response to IFN-γ
Source: Mol Cell Proteomics. 2021 Sep 28;21(4):100153. doi: 10.1016/j.mcpro.2021.100153 (PMC9014395; doi:10.1016/j.mcpro.2021.100153)
Supplement: Supplemental Figures S1–S11 [file mmc1.pdf]

Supplemental Figures

A Novel Spectral Annotation Strategy Streamlines Reporting of mono-ADP-ribosylated Peptides Derived from Mouse Liver and Spleen in Response to IFN-γ

Shiori Kuraoka1, Hideyuki Higashi, Yoshihiro Yanagihara, Abhijeet R. Sonawane, Shin Mukai, Andrew K. Mlynarchik, Mary C. Whelan, Michael O. Hottiger, Waqas Nasir, Bernard Delanghe, Masanori Aikawa1 and Sasha A. Singh

LIST OF SUPPLEMENTAL FIGURES

Supplemental Figure S1. Removal of GST-tagged-eAf1521 protein from ADPr-peptides using various commercially available molecular weight cut-off (MWCO) filters.
Supplemental Figure S2. Molecular weight cut-off (MWCO) filters impact HeLa peptide / proteome recovery.
Supplemental Figure S3. Instrument acquisition methods and samples analyzed.
Supplemental Figure S4. Proteome Discoverer 2.4 exports of a mouse liver ADPr peptide analyzed by HCD, CID or ETD on the Lumos**.**
Supplemental Figure S5. The *m*-ion and *p*-ion series score distributions at varying HCD and CID collision energies.
Supplemental Figure S6. Individual precursor *P*-ion distributions at select HCD or CID collision energies.
Supplemental Figure S7. The effect of precursor charge on XCorr vs. p-series scores.
Supplemental Figure S8. Mouse liver and spleen respond to IFN-g injection.
Supplemental Figure S9. Mouse liver and spleen anti-ADP-ribosylation Western blots analysis.
Supplemental Figure S10. Validation of a PARP14 ADPr peptide from Figure 7.
Supplemental Figure S11. Rank2 spectrum is correct for FABP1 in Figure 7.

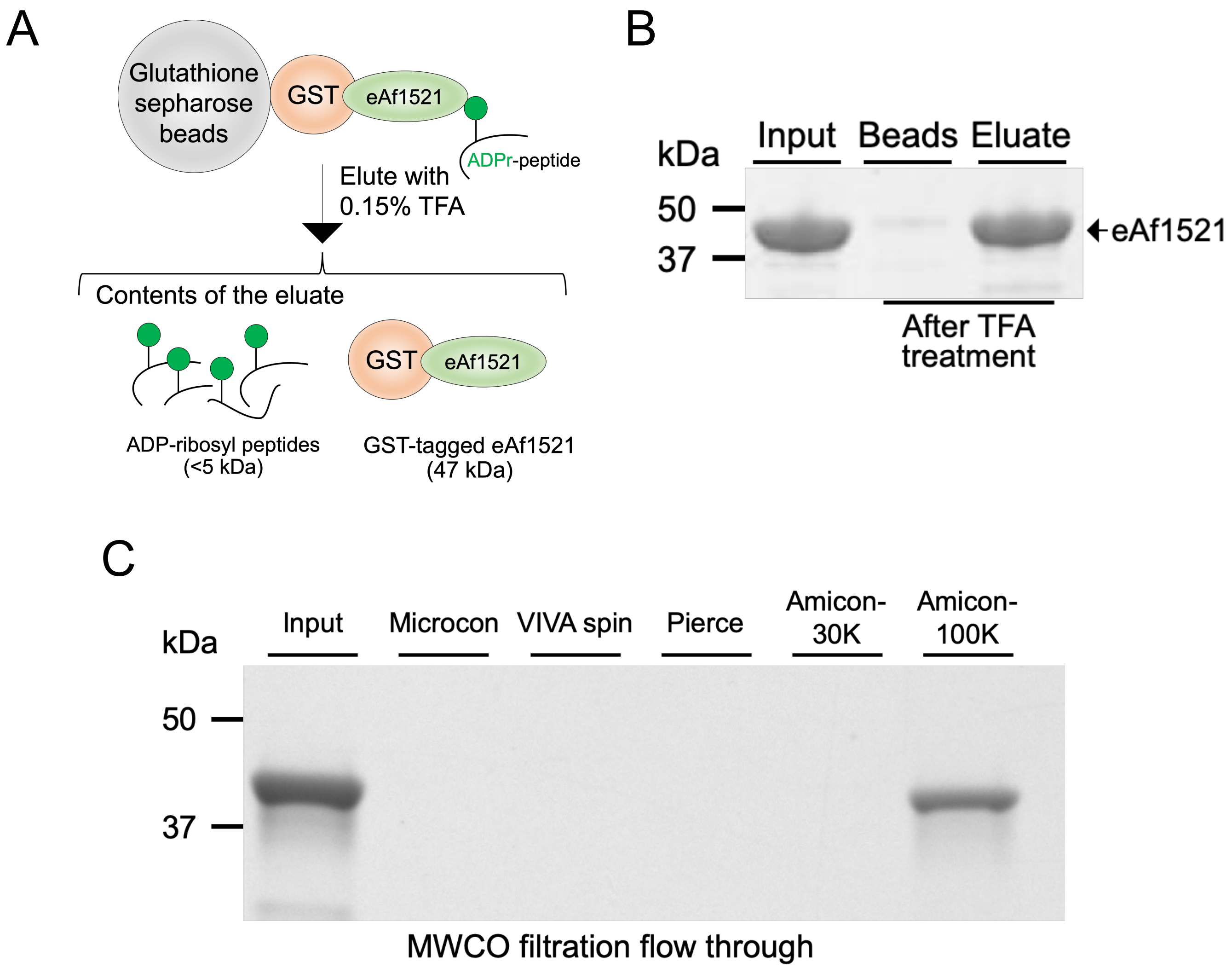

Supplemental Figure 1. Removal of GST-tagged-eAf1521 protein from ADPr-peptides using various commercially available molecular weight cut-off (MWCO) filters*. *A*, The final step for the prototypical ADPr-peptide enrichment protocol is the trifluoroacetic acid (TFA) release of ADPr-peptides and eAf1521 from sepharose beads. *B*, A coomassie blue stain of the ADPr-peptide eluate that still harbors eAf1521 protein. *C*, MWCO flow-throughs of unbound, free eAf1521. The Amicon-100K filter was a filter control where it was expected that eAf1521 would not be retained.

*eAf1521 (47 kDa) removal was tested using five molecular weight cut off (MWCO) filter cartridges: 1) Microcon-30 kD Centrifugal Filter Unit Millipore Sigma, Cat# MRCF0R03; 2) Pierce Protein Concentrators PES, 30K MWCO, Thermo Fishe Scientific, Cat# 885022; 3) Amicon Ultra-0.5 Centrifugal Filter Unit, Millipore Sigma, Cat# UFC5030243; 4) Amicon Ultra-0. Centrifugal Filter Unit, 100 kDa, Millipore Sigma, Cat# UFC510024; and 5) Vivaspin 500, 30K MWCO PES (Sartoriu Göttingen, Germany, Cat # VS0121). MWCO cartridges 1 to 4 were equilibrated by passing 300 µL of 20% acetonitrile (Fishe Scientific, Cat# A955-1) /LC/MS-grade water (Fisher Scientific, Cat# W6-1) twice, 300 µL of 0.1 mol/L NaOH (Honeywe International, Charlotte, NC, Cat# 71463 Fluka) twice, 300 µL of LC/MS-grade water twice, and 300 µL of 0.15% trifluoroacet acid (TFA, Sigma Aldrich, Cat# 302031) three times (14,000 rpm, 5 minutes).

Details on the generation of eAf1521 variant are detailed here: Nowak *et al*., *Nat Comm*. 2020 (PMID: 33060572).

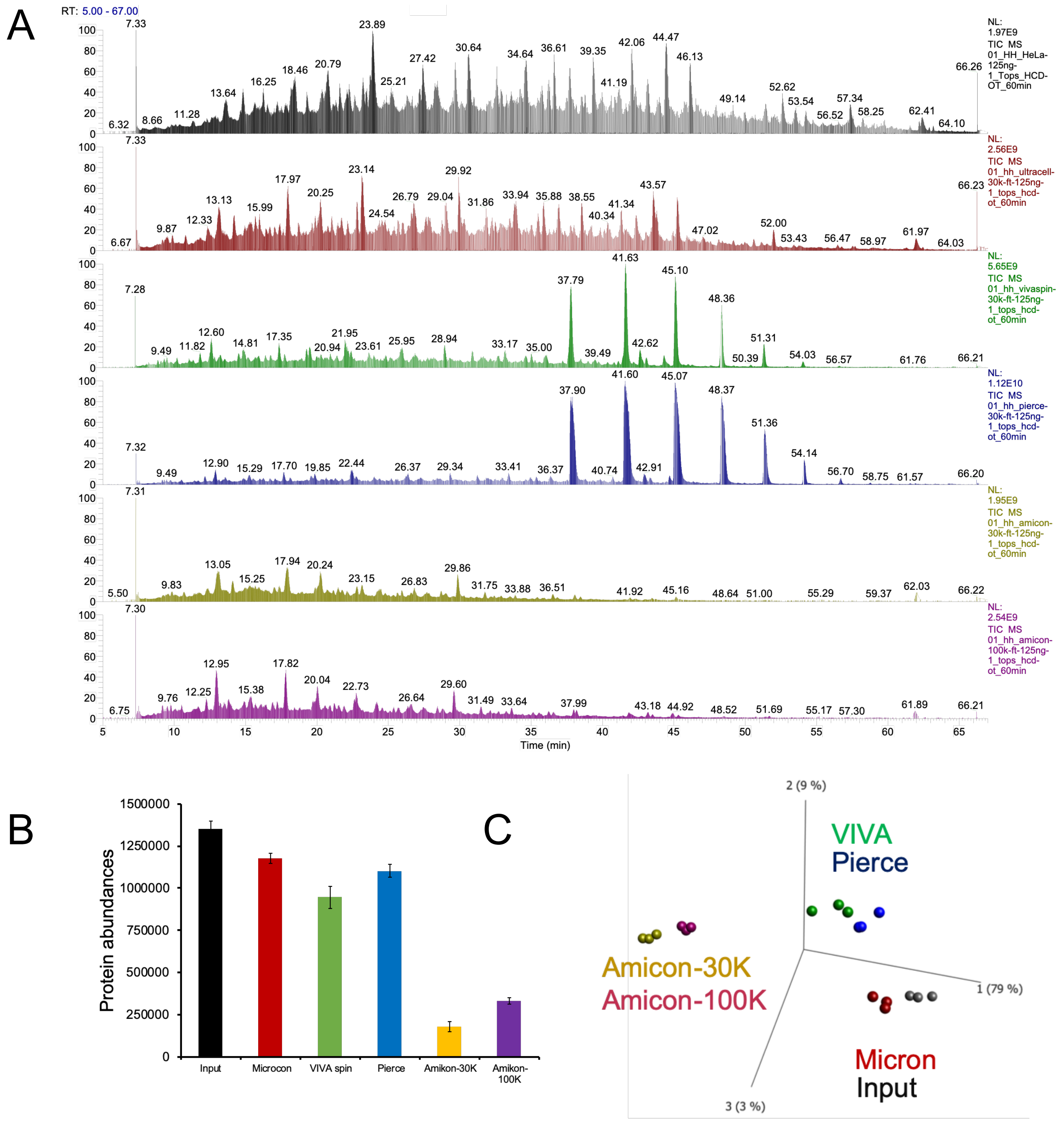

Supplemental Figure S2. Molecular weight cut-off (MWCO) filters impact HeLa peptide / proteome recovery. *A*, Representative total ion current (TIC) chromatograms from each peptide sample type. *B*, The average normalized protein abundances for each MWCO workflow. *C*, Principle component analysis of the resulting proteome data demonstrating the the Micron (UltraCell) filter most resembles the input peptide sample.

Collision energy study

Instrument	Sample	Fragmentation method	Number of files
Lumos	Mouse liver (pooled pilot samples)	HCD (CE: 20, 24, 26, 28, 30, 32, 34%)	7 (1 sample, one injection per collision energy)
		CID (CE: 20, 24, 26, 28, 30, 32, 34, 36, 40%)	9 (1 sample, one injection per collision energy)
Q Exactive	Mouse liver (pooled pilot samples)	HCD (CE: 20, 24, 26, 28, 30, 32, 34%)	7 (1 sample, one injection per collision energy)

Main study (ADPr proteins)

Instrument	Sample	Fragmentation method	Number of files
Lumos	Mouse liver (Control, Saline, IFN-γ; n=6 per group)	m-ion triggered HCD (CE: 25, 27.5, 30%) + EThcD (22.5%)	90 (18 samples, five GPS injections per sample)
	Mouse spleen (Control, Saline, IFN-γ; n=3 per group)	m-ion triggered HCD (CE: 25, 27.5, 30%) + EThcD (22.5%)	45 (9 samples, five GPS injections per sample)
GPS	*m/z* 400-1500, 400-600, 600-800, 800-100,1000-1200		

Main study (proteomes)

Instrument	Sample	Fragmentation method	Number of files
Lumos	Mouse liver (Control, Saline, IFN-γ; n=6 per group)	Data-dependent HCD acquisition (CE: 27.5, 30, 32.5%)	18 (18 samples, one injection per sample)
	Mouse spleen (Control, Saline, IFN-γ; n=3 per group)	Data-dependent HCD acquisition (CE: 27.5, 30, 32.5%)	9 (9 samples, one injection per sample)

Targeted MS for PARP14 ADPr peptide

Instrument	Sample	Fragmentation method	Number of files
Lumos	Mouse spleen (pooled pilot samples)	m/z 555.9058 (z=3) targeted HCD (CE: 20 or 22%)	1 (1 sample, one injection)
	Mouse spleen (pooled pilot samples)	m/z 555.9058 (z=3) targeted HCD (CE: 25, 28, 31%) + EThcD (22.5%)	1 (1 sample, one injection)

Supplemental Figure S3. Instrument acquisition methods and samples analyzed.

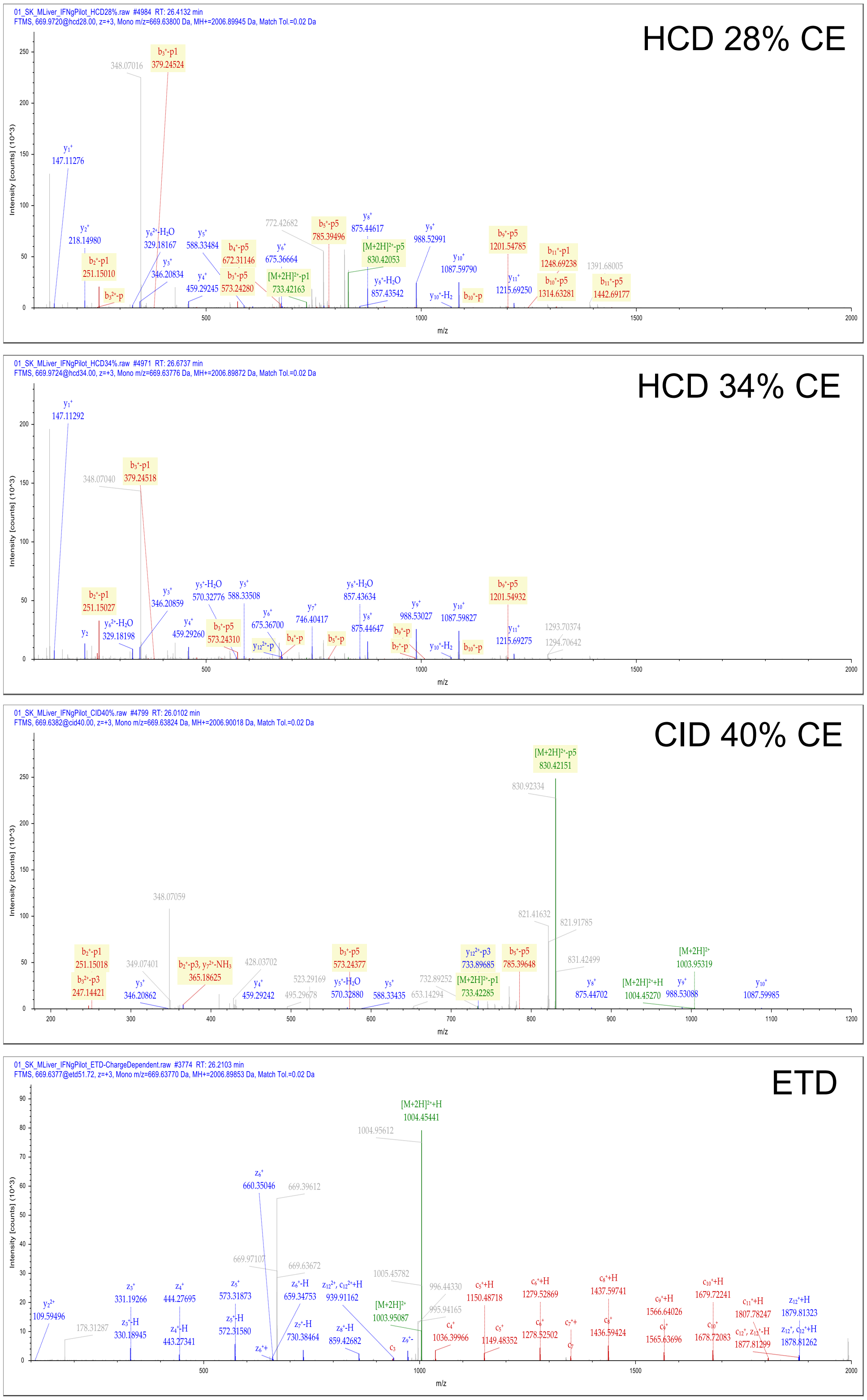

Supplemental Figure S4. Proteome Discoverer 2.4 exports of a mouse liver ADPr peptide analyzed by HCD, CID or ETD on the Lumos. The HCD and CID SELENBP1/2 ADPr spectra are also in Figure 2. All fragment ions (*b*/*y*-ion and *p*-ion series) except the *m*-ions are annotated in PD2.4.

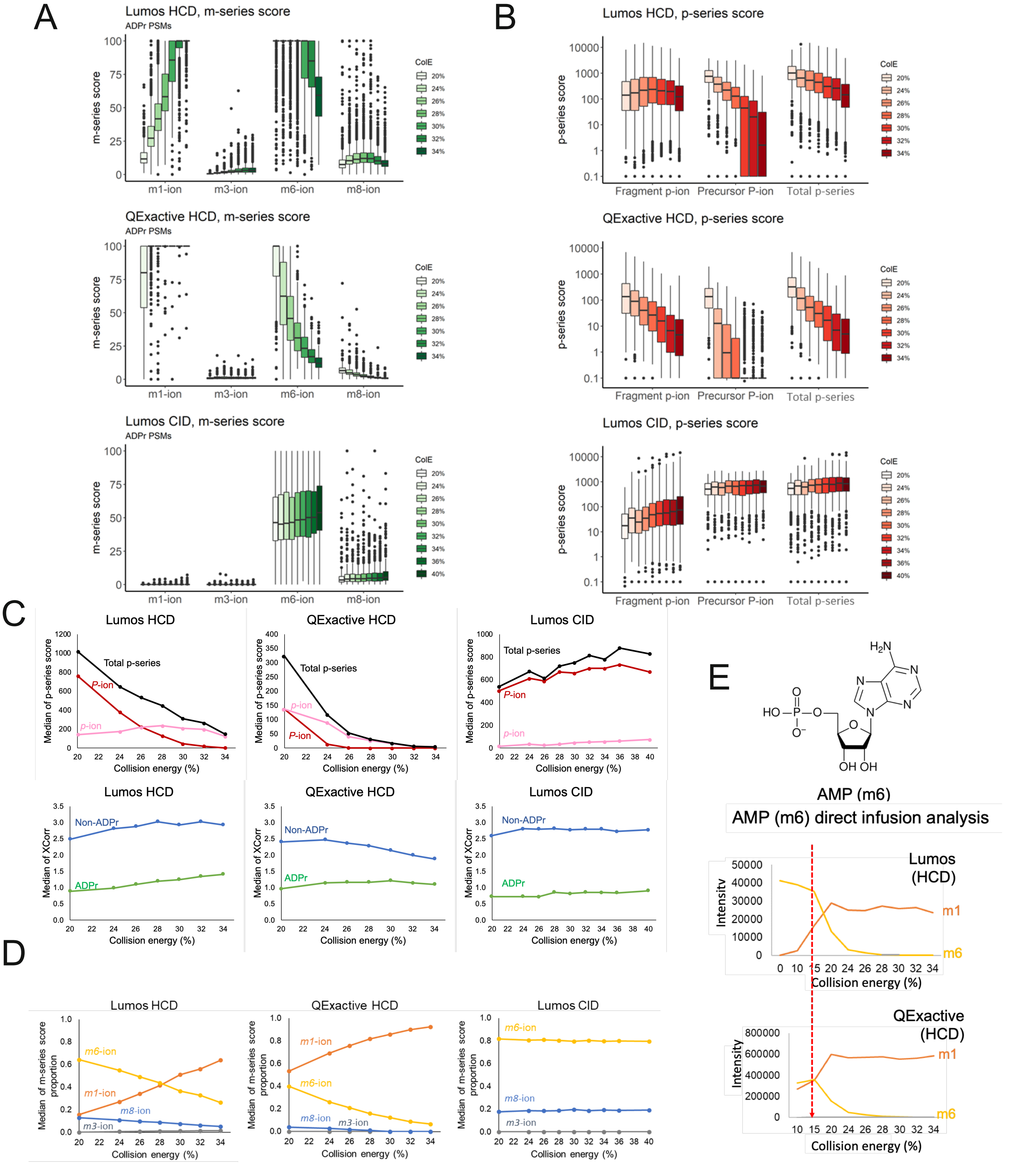

Supplemental Figure S5. The *m*-ion and *p*-ion series score distributions at varying HCD and CID collision energies. *A*, *m*-ion score breakdown for each instrument and collision energy. B, Fragment *p*-ion, precursor *P*-ion and the total p-series scores distributions for each instrument and collision energy. C, The various p-series scores (median) vs. collision energy% and the XCorr (median) vs. collision energy% for non-ADPr and ADPr spectra identified from MARylated peptide samples. *D*, The individual *m*-ion score proportions (median) vs. collision energy%. *E*, Direct infusion of AMP (m6) demonstrating conversion to adenine (m1) with HCD performed on the Lumos or Q Exactive.

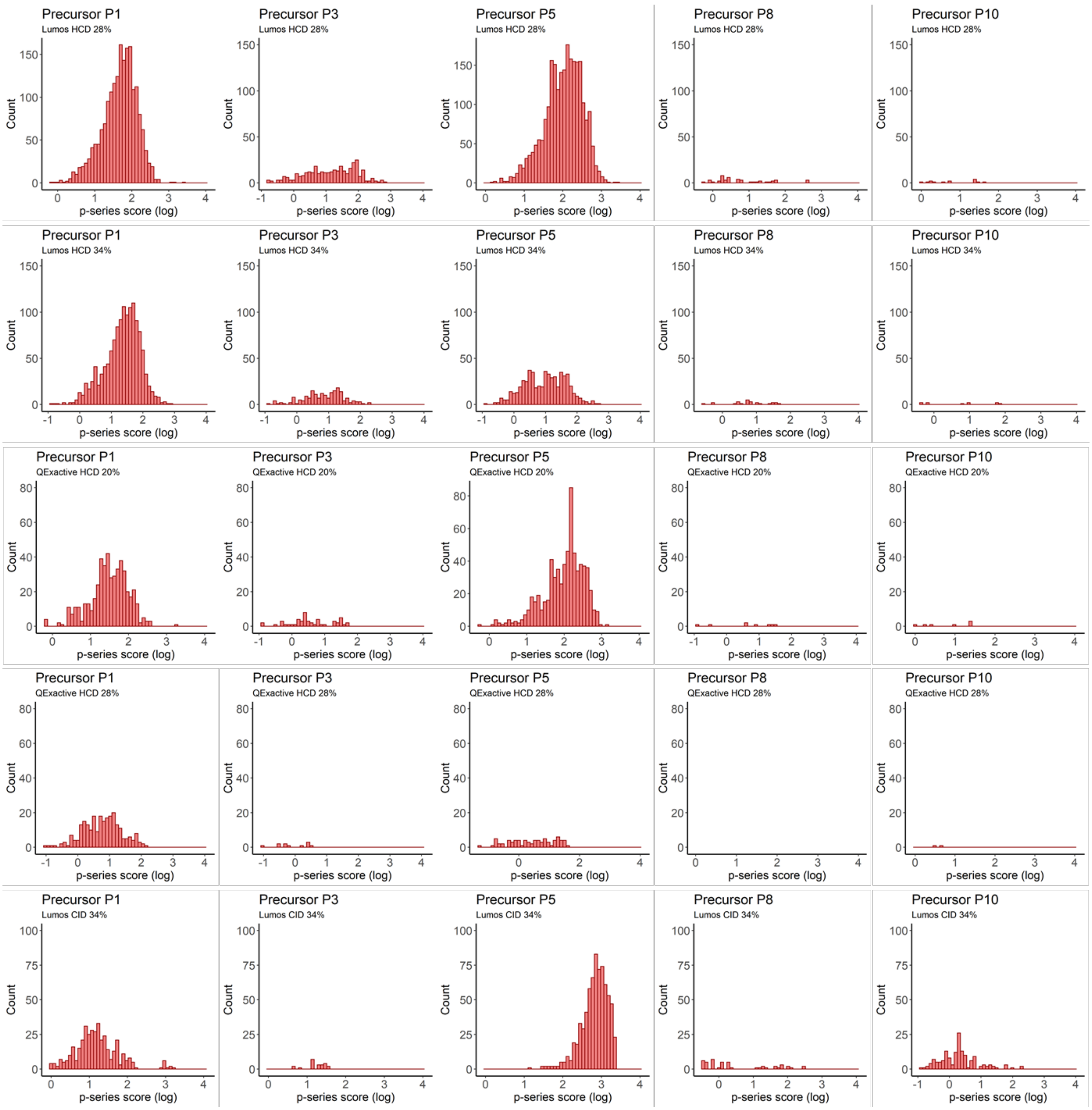

Supplemental Figure S6. Individual precursor *P*-ion distributions at select HCD or CID collision energies. Histograms are 50-bins.

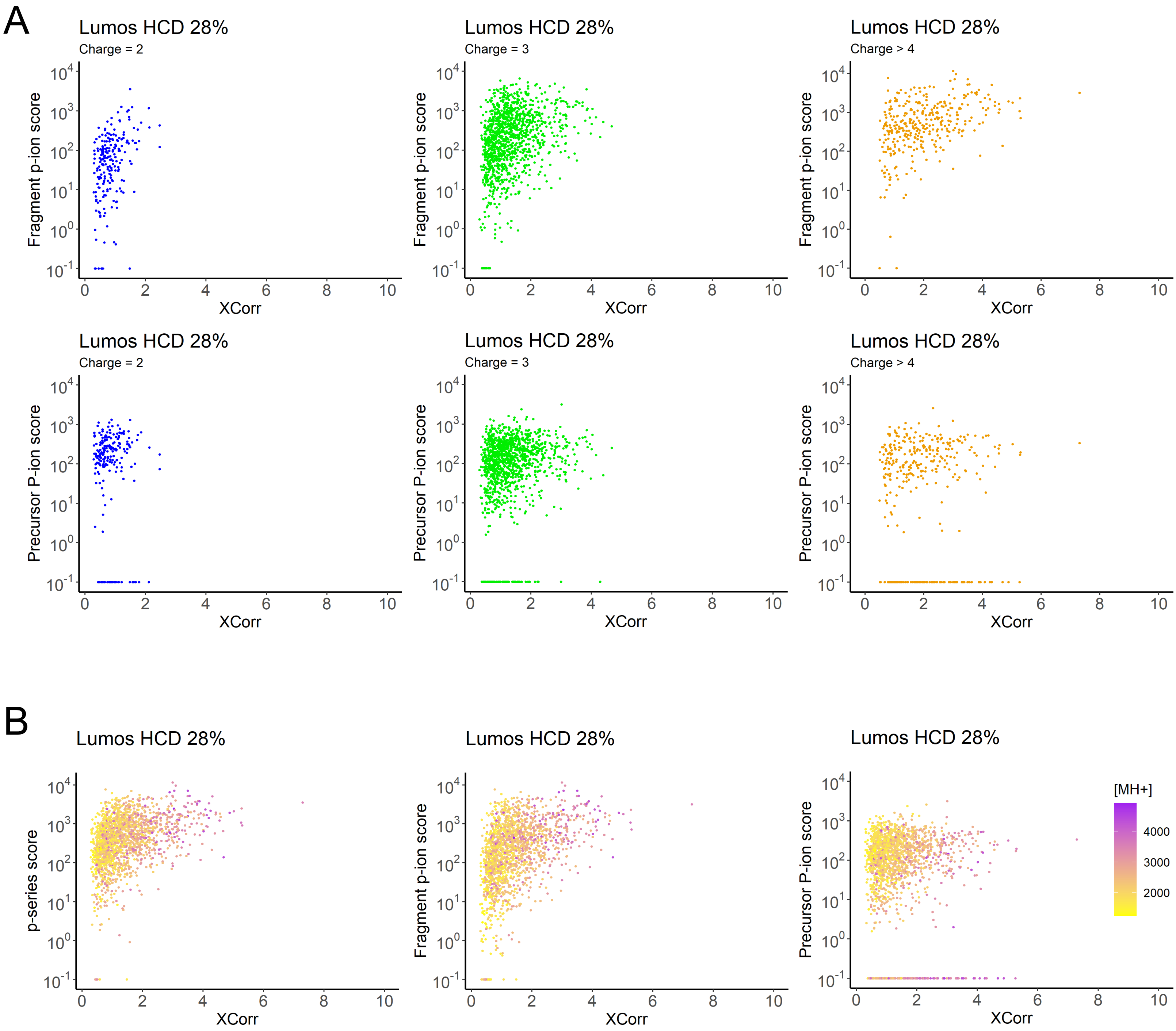

Supplemental Figure S7. The effect of precursor charge on XCorr vs. p-series scores. *A*, Fragment p-ions (upper panels) or precursor P-ions (lower panels) *B*, The analysis of precursor mass [MH+] distribution when the p-series scores are compared to XCorr.

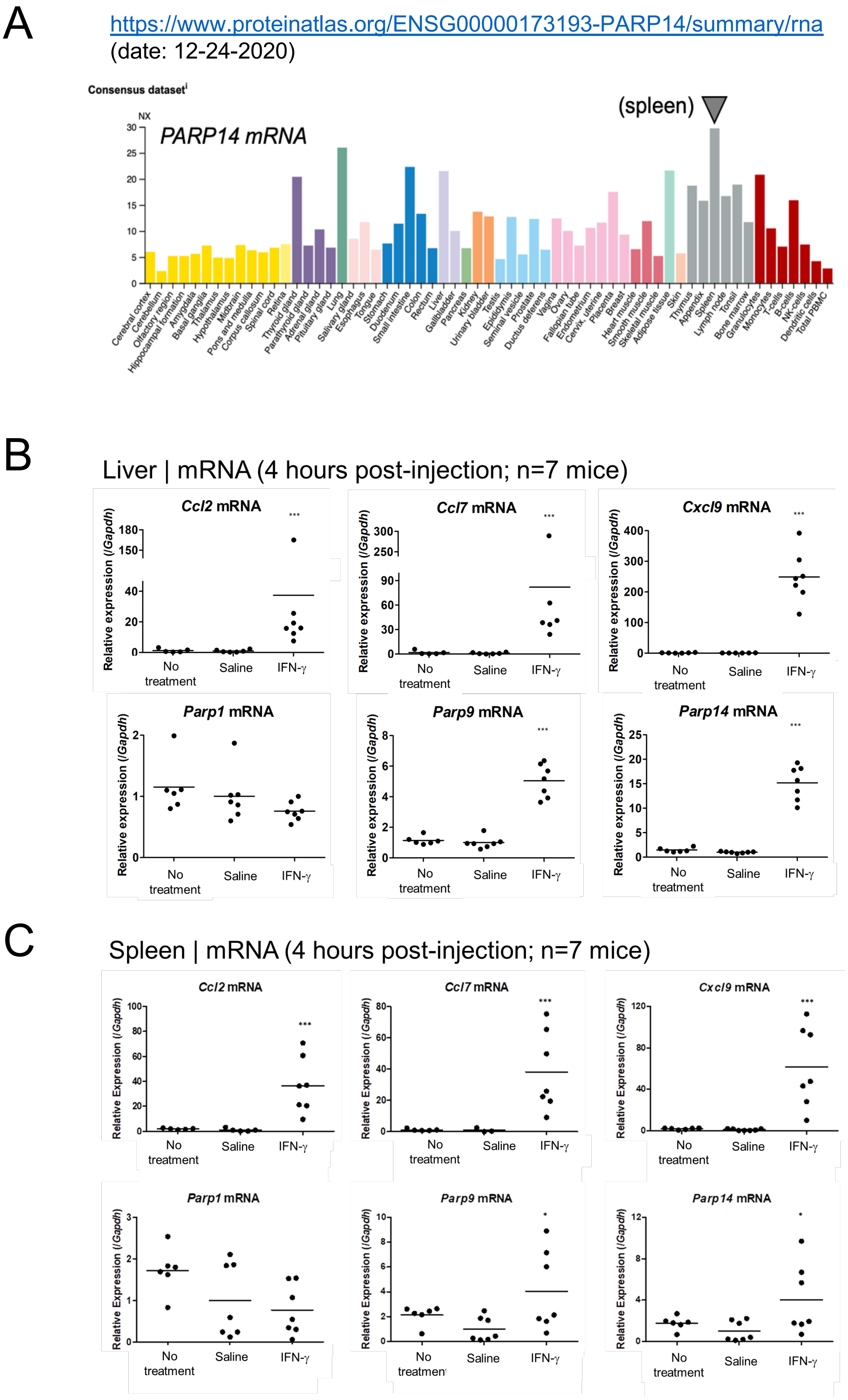

Supplemental Figure S8. Mouse liver and spleen respond to IFN-γ injection. *A*, *PARP14* mRNA expression profile on the human protein atlas database. *B*, *C*, Cytokines (upper panels) and *PARPs* mRNA responses to 4 hours IFN-γ: 100 K units/mouse for liver (B) and spleen (C). Saline expression = 1.0. t-test with Welch's correction; Saline vs IFN-γ. ***$p<0.001$, **p<0,01, *$p<0.05$.

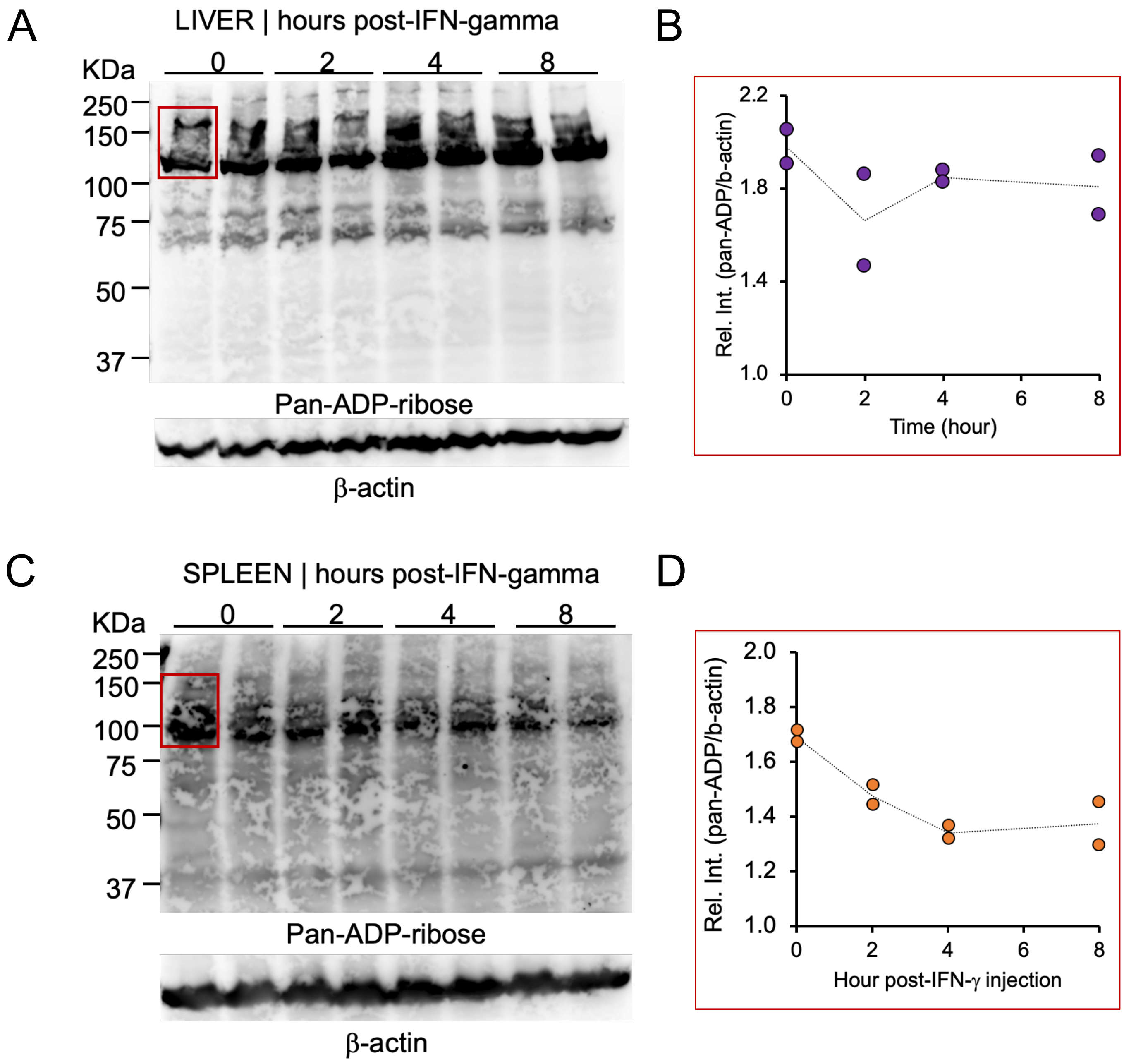

Supplemental Figure S9. Mouse liver and spleen anti-ADP-ribosylation Western blots analysis. *A*, *B*, liver and *C*, *D*, spleen. N=2 mice organs per time point. The red box in the gel corresponds to the region used for quantification by ImageJ (B,D).

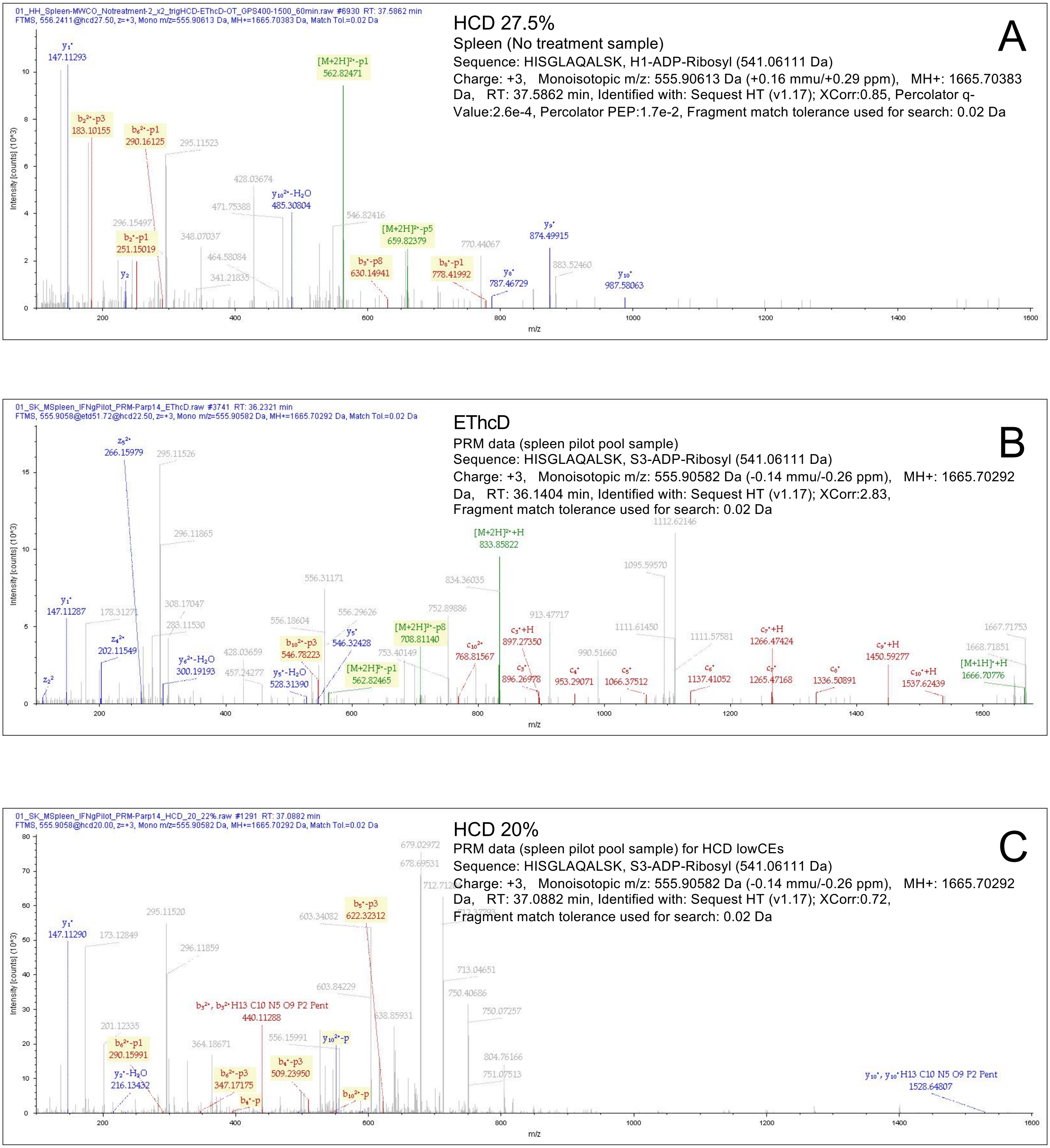

Supplemental Figure S10. Validation of a PARP14 ADPr peptide from Figure 7. *A*, HCD spectrum derived from a spleen no treatment control sample indicating histidine as the acceptor site *B* and *C*, PRM scans from EThcD (B) and HCD 20% CE (C) reveal serine to be the correct acceptor site and not histidine.

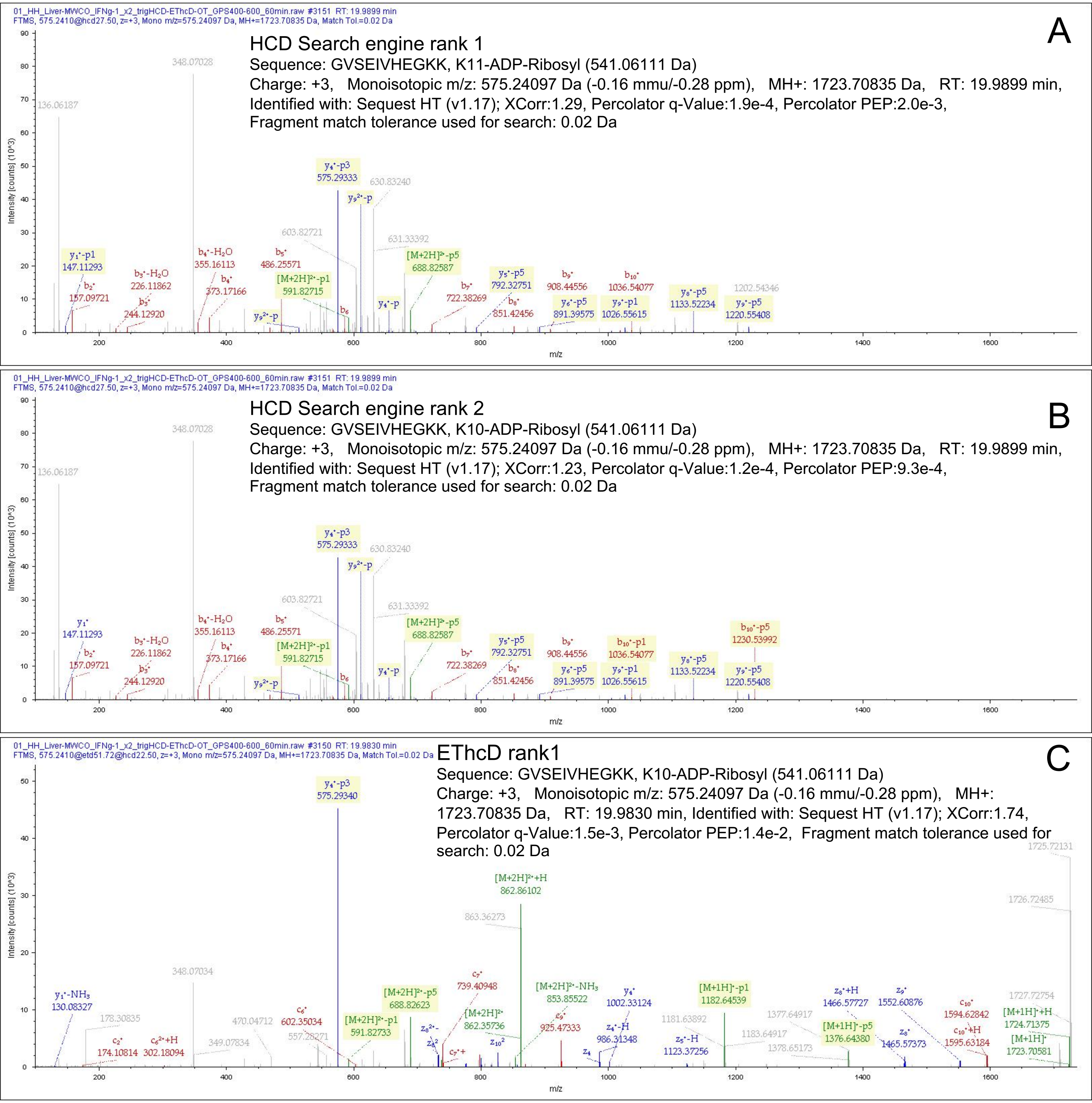

Supplemental Figure S11. Rank2 spectrum is correct for FABP1 in Figure 7. *A* and *B*, the same HCD scan with SEQUEST-HT rank1 and rank2 acceptor site assignments. *C*, The corresponding EThcD scan.
